# Massive over-representation of solute-binding proteins (SBPs) from the tripartite tricarboxylate transporter (TTT) family in the genome of the α-proteobacterium *Rhodoplanes* sp. Z2-YC6860

**DOI:** 10.1099/mgen.0.000176

**Published:** 2018-04-18

**Authors:** Leonardo T. Rosa, Vicki Springthorpe, Matheus E. Bianconi, Gavin H. Thomas, David J. Kelly

**Affiliations:** ^1^​ Department of Molecular Biology and Biotechnology, The University of Sheffield, Sheffield, UK; ^2^​ Department of Biology, Wentworth Way, University of York, York, UK; ^3^​ Department of Animal and Plant Sciences, The University of Sheffield, Sheffield, UK

**Keywords:** Solute transporter, periplasmic-binding protein, Gene duplication, gene over-representation, lineage specific expansion

## Abstract

Lineage-specific expansion (LSE) of protein families is a widespread phenomenon in many eukaryotic genomes, but is generally more limited in bacterial genomes. Here, we report the presence of 434 genes encoding solute-binding proteins (SBPs) from the tripartite tricarboxylate transporter (TTT) family, within the 8.2 Mb genome of the α-proteobacterium *Rhodoplanes* sp. Z2-YC6860, a gene family over-representation of unprecedented abundance in prokaryotes. Representing over 6 % of the total number of coding sequences, the SBP genes are distributed across the whole genome but are found rarely in low-GC islands, where the gene density for this family is much lower. This observation, and the much higher sequence identity between the 434 *Rhodoplanes* TTT SBPs compared with the average identity between homologues from different species, is indicative of a key role for LSE in the expansion. The TTT SBP genes were found in the vicinity of genes encoding membrane components of transport systems from different families, as well as regulatory proteins such as histidine-kinases and transcription factors, indicating a broad range of functions around the sensing, response and transport of organic compounds. A smaller expansion of TTT SBPs is known in some species of the β-proteobacteria *Bordetella* and we observed similar expansions in other β-proteobacterial lineages, including members of the genus *Comamonas* and the industrial biotechnology organism *Cupriavidus necator*, indicating that strong environmental selection can drive SBP duplication and specialisation from multiple evolutionary starting points.

**Impact Statement**

Intraspecific gene homology is a common evolutionary feature among prokaryotes, helping to build metabolic versatility and adaptability, but is mostly restricted in terms of gene numbers. Here, the over-representation of a gene family of unprecedented magnitude in a prokaryotic genome is described, with 434 genes belonging to the tripartite tricarboxylate transporter solute-binding proteins family in the α-proteobacterium *Rhodoplanes* sp. Z2-YC6860 genome. Although over-representations of smaller scale have been characterised for this family in some β-proteobacteria, we provide evidence that lineage-specific expansion, rather than horizontal gene transfer, is likely to be the major driving mechanism for this expansion, indicating that environmental selection can drive the expansion of binding proteins independently in multiple organisms.

## Introduction

Gene family expansions result from events of gene duplication and horizontal gene transfer (HGT), and are major drivers for metabolic versatility and adaptation in prokaryotes and eukaryotes [1–3]. The results of high-throughput analyses of prokaryotic genomes indicate that the number of homologous genes correlates positively with genome size, possibly reflecting the versatility required from the complex environments that bacteria with large genome sizes often inhabit [1, 4–7], and that species showing similar families of homologues usually show similar metabolic features [1]. However, although the sum of genes encoding proteins that form members of gene families is significant, the number of members of individual families is usually low, with only 2 % of such families showing three or more copies [6–8], and there are very few cases where an over-representation of a single gene family is observed in a prokaryotic genome that is not related to transposon and phage insertions [6].

One of the few exceptions is the observed over-representation of histidine kinases (HPKs), reaching over 100 gene copies and representing 2.5 % of the coding sequences in some genomes [9, 10]. Although HGT seems to have played an important role in the expansion of this gene family, the largest over-representations are a consequence of gene duplication leading to lineage-specific expansions (LSE), which then results in the occurrence of ‘orphan’ HPKs (i.e. not encoded next to their cognate response regulators), that subsequently diverge through rearrangements in their N-terminal signalling domain to sense different environmental conditions [9]. In genomes where the expansion is driven by HGT, the HPK and the response regulators are duplicated together, undergoing a rapid selective pressure to co-evolve and cease ‘cross-talk’ with other systems [11].

A second example is the genome of the subsurface α-proteobacterium *Geobacter sulfurreducens,* which contains 111 genes encoding proteins of the cytochrome *c* family, representing 3 % of the total theoretical proteome [12]. This over-representation correlates with the versatile bioenergetics of this organism, which uses metal-ion-mediated electron transport to oxidize its substrates [12], although the actual function of the vast majority of these cytochromes is unknown.

A third example is the genome of the gut symbiotic *Bacteroides thetaiotaomicron,* which revealed the existence of 106 homologous genes to *susC* and 57 homologous genes to *susD*, encoding components of outer membrane uptake systems. In addition, 71 genes encoding glycosyltransferases are present, being involved in capsular polysaccharide biosynthesis. The over-representation of these two families is believed to relate both to the versatile uptake and utilisation of otherwise non-digestible sugars and evasion of the human immune system [10].

A final example of a large gene over-representation in bacterial genomes, relates to solute-binding proteins (SBPs) of the tripartite tricarboxylate transporters (TTT) family [13]. The TTT family are secondary transporters that use a periplasmic SBP for initial substrate recognition and binding. The TctCBA citrate uptake system from *Salmonella typhimurium* is the prototypical transporter of this family [14]. Each complete system is composed of three subunits: a 12-helix transmembrane protein, homologous to TctA and thought to be the solute carrier itself, a poorly-conserved four-transmembrane-domain accessory protein, homologous to TctB and of unknown function, and a periplasmic SBP, homologous to TctC and responsible for substrate specificity and initial high-affinity binding.

Genomic studies in the whooping-cough-causing agent *Bordetella pertussis* revealed the existence of 79 genes encoding TTT SBPs [15], and further analysis revealed that this over-representation was found in many species of the genus *Bordetella*, reaching 181 genes in *Bordetella bronchiseptica,* and in a closely related bacterium, *Cupriavidus metallidurans* [16]. Intriguingly, the number of genes for the membrane components did not follow the same pattern, showing no more than four representatives in *Bordetella*, leaving the majority of the SBPs as ‘orphans’. Later genome releases indicated that this over-representation was found also in other β-proteobacteria of the genus *Advenella* [17]. In this study, we further analyse the distribution of TTT SBPs in bacteria and discover a group of α-proteobacteria that have independently expanded their use of these proteins to unprecedented levels for any gene family in any currently known bacterial genome.

## Results and discussion

**Fig. 1.**
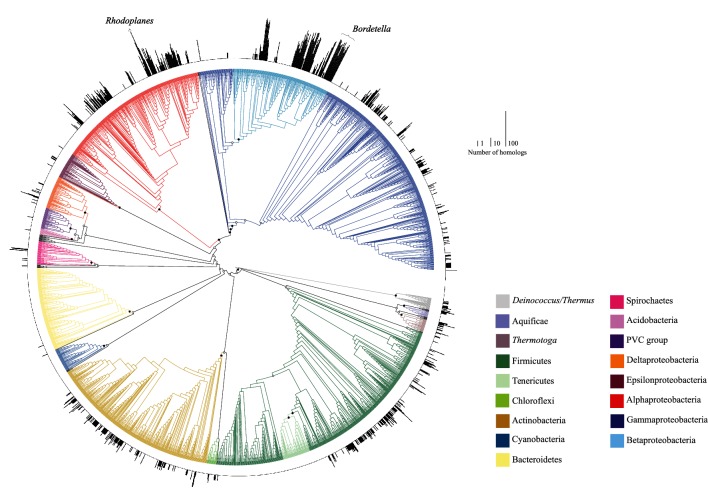
Distribution of TTT SBPs in the genomes of bacteria. The outer circle represents the number of TTT SBPs present in each genome, using a log_2_ scale. The tree was inferred using 16S rRNA sequences retrieved from the genome of each organism, and aligned using MAFFT v7 [37]. A maximum-likelihood tree was inferred using RAxML v8.2.11 [38] under the GTRCAT model, with 100 bootstrap pseudoreplicates. Bootstrap support values are indicated on nodes of major lineages when higher than 50 % (inclusive; filled circles) or lower than 50 % (open circle). Major branches are coloured as indicated in the key. Non-coloured branches are minor lineages of Bacteria.

While studying the function and diversity of TTT transporters [13, 18], we analysed the number of TTT SBP homologues encoded amongst the available bacterial genomes, as previously described [13]. As of November 2017, 8049 fully assembled bacterial genomes were available in the NCBI database, belonging to 2323 different species. We used one genome per species for the analysis below. A homology search was performed using the TBLASTN tool with an e-value cut-off of 10^−15^, with the following set of functionally characterised TTT SBPs as query: TctC [19], BugD [20], BugE [21], Bug27 [22], TphC [23] and AdpC [18]. The resulting data were mapped onto a 16S rRNA tree to determine how the frequency of the SBPs correlated with the phylogenetic position of the organisms (Fig. 1). The known expansions of the TTT SBPs in the *Bordetellae* [16] can be clearly identified in the tree, as well as other expansions within the β-proteobacteria. However, examples of genomes enriched in TTT SBPs can also be seen in the α-proteobacteria, and most strikingly in the genome of *Rhodoplanes* sp. Z2-YC6860 (Assembly: GCA_001579845.1), where 434 TctC homologs were found to be encoded in its 8.2 Mbp genome, representing 6.2 % of the total coding sequences (CDS) (Fig. 1). Other closely related α-proteobacteria show similar but smaller enrichments, including *Pseudorhodoplanes sinuspersici* RIPI110^T^, with 99 TTT SBPs, and *Bradyrhizobium icense* LMTR 13^T^, with 43 TTT SBPs.

**Fig. 2.**
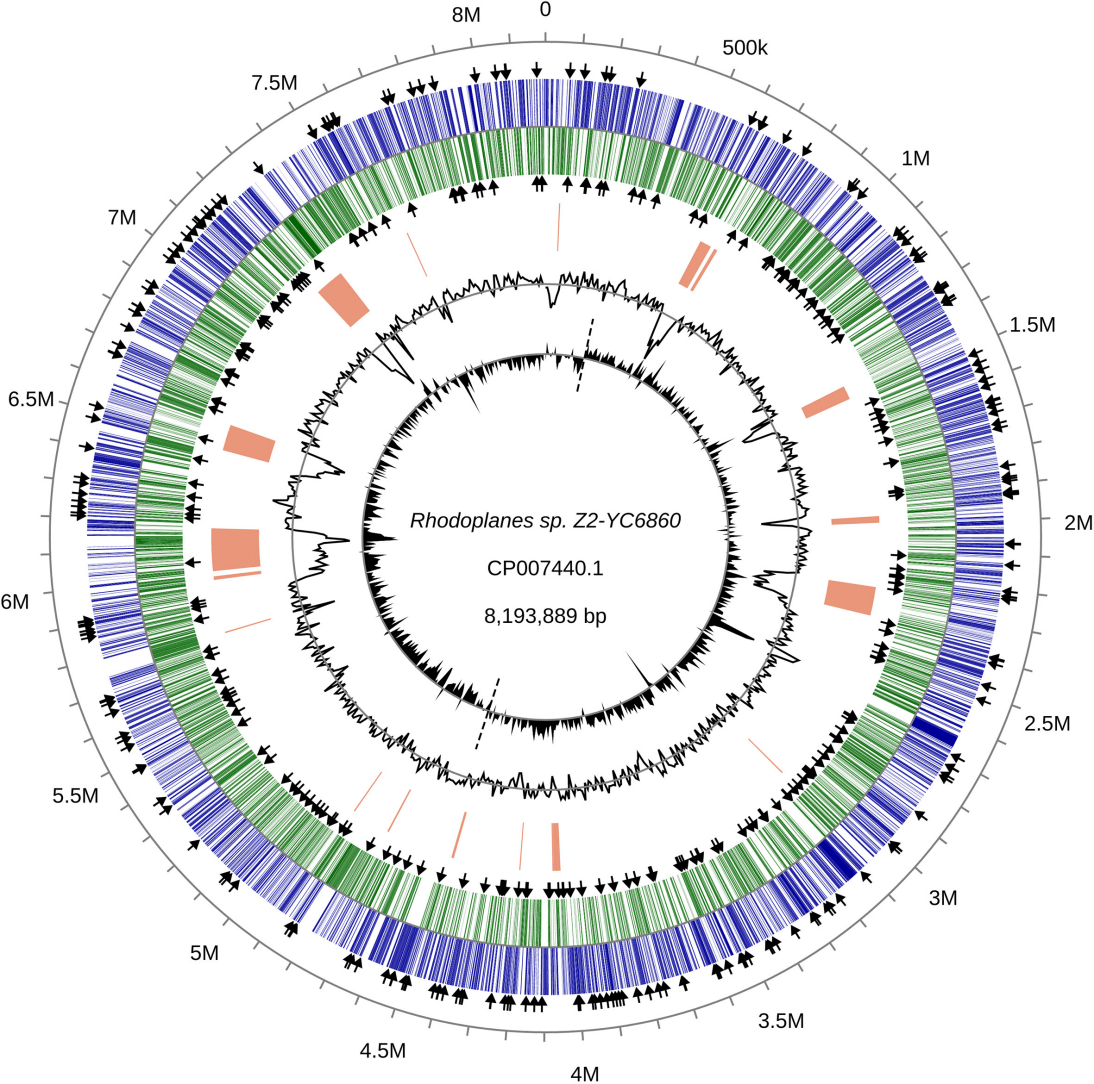
Circular genome plot of the 8.193 Mb *Rhodoplanes* sp. Z2-YC6860 genome. The outer two tracks represent CDS on the forward strand (blue) and reverse strand (green), with black arrows indicating the location of TTT SBP genes. Orange blocks indicate genomic islands predicted by IslandViewer software [25]. The next inner circular plot represents percentage GC content calculated over a 10 kb sliding window with a range of 52.4 to 69.1% and mean of 63.5 %. The innermost circular plot represents GC skew calculated as (G−C)/(G+C) over a 10 kb sliding window. The characteristic GC skew reversal at the origin and terminus of replication is indicated by dashed lines.

We examined the genome of *Rhodoplanes* sp. Z2-YC6860 in more detail to check the quality of the assembly and to investigate where the genes for the 434 TTT SBPs were located (Fig. 2). To check the assembly we plotted the GC skew (innermost ring in Fig. 2) and observed the typical skew reversal from the likely origin of replication until the likely termination site [24]. We also plotted the GC content (next innermost ring in Fig. 2) and could identify likely regions of DNA with atypically low GC contents, which were also predicted to be genomic islands using IslandViewer4 [25]. Interestingly, although the TTT SBP genes are distributed across the whole genome, the number of such genes in these low-GC regions, which comprise 9.9 % of genome length, with a GC content of 58.1 %, is only 11, which gives a ‘density’ of TTT SBP genes in these regions of 13.5 genes per Mb. The rest of the genome, in contrast, has a GC content of 64.1 %, and contains 423 TTT SBP genes, so has a ‘density’ of 57.3 TTT SBP genes per Mb. This striking tenfold difference in density indicates that recent detectable HGT is not the primary route by which these genes have been acquired as these regions in effect dilute the density of the TTT SBPs found in the rest of the genome.

Examination of the arrangement of the TTT SBPs on the genome reveals that there are 48 sets of these genes arranged in tandem arrays, while others are present in longer arrays of three, four and five genes (Table 1). Remarkably, there is a single array containing nine TTT SBPs adjacent to each other. The closely related *Pseudorhodoplanes sinuspersici* RIPI110^T^ and *Bradyrhizobium icense* LMTR 13^T^ also show evidence of tandem arrays, but not of this complexity. However, we cannot exclude the presence of similar organisations in closely related bacteria where genomic information is not available.

**Table 1.** Occurrence of TTT SBP genes as single genes or in larger arrays of genes, in the genome of *Rhodoplanes* sp. Z2-YC6860 and two closely related bacteria

Array size (number of genes)	Frequency		
	*Rhodoplanes* sp. Z2-YC6860	*Pseudorhodoplanes sinuspersici* RIPI110^T^	*Bradyrhizobium icense* LMTR 13^T^
1	294	93	39
2	48	3	2
3	6	–	–
4	3	–	–
5	1	–	–
6	–	–	–
7	–	–	–
8	–	–	–
9	1	–	–

To investigate the mechanisms driving this massive over-representation, each of the 434 TTT SBP’s protein sequences of *Rhodoplanes* sp. Z2-YC6860 was searched using the blastp tool against a database comprising all TTT SBP’s identified in all 2323 bacterial genomes (see above). Self-matches were excluded and only the best hit (highest identity) was reported (Table S1, available in the online version of this article). Out of the 434 TTT SBP’s of *Rhodoplanes* sp. Z2-YC6860, a total of 234 sequences (53.9 %) had the best hit within the same genome. For another 98 sequences (22.6 %), the best hit was found in representatives of Rhizobacteria, which is the α-proteobacteria group that includes the genus *Rhodoplanes*. The best hit of the remaining 102 sequences (23.5 %) was found in species that are not within the α-proteobacteria group (Table S2). These findings suggest that gene duplications within *Rhodoplanes* sp. Z2-YC6860 account for more than half of this species’ TTT SBP’s, pointing to a key role of LSE in this unprecedented protein family expansion. The fact that another 22.6 % of the total genes have their best hit in other representatives of Rhizobacteria is indicative of duplication events preceding the origin of *Rhodoplanes*. Such best hits probably represent orthologous genes in these closely related species. The 23.5 % remaining genes with the best hit in non-α-proteobacteria species would probably have originated via HGT, although in this case it is unclear whether *Rhodoplanes* would have acted as donor or recipient of these genes. Note however, that a comprehensive phylogenetic analysis of the TTT SBP protein sequences across the Proteobacteria would be necessary to distinguish among these scenarios, and to exclude the hypotheses of (1) multiple gene losses in closely related groups [26], which could also produce the over-representation pattern that was exclusive to *Rhodoplanes* sp. Z2-YC6860, and/or (2) convergent sequence evolution, which could lead to higher sequence identity between distantly related species, therefore masking the actual mechanism underlying the origin of the gene [27].

**Fig. 3.**
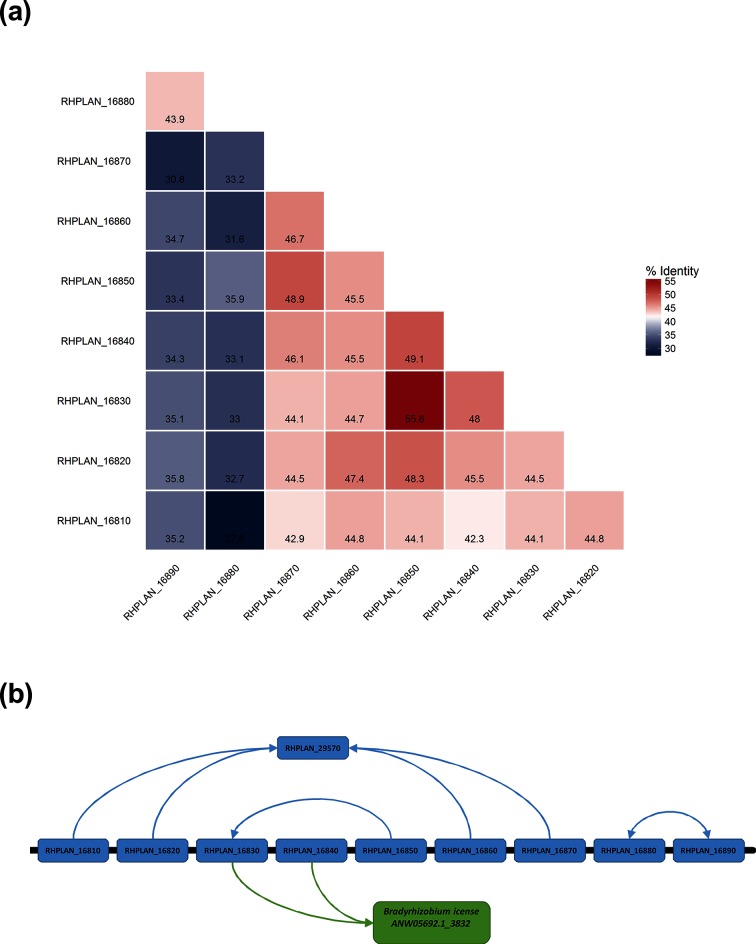
Analysis of the relationships between the nine TTT family proteins located in the largest tandem array found in *Rhodoplanes* sp. Z2-YC6860. (a) Similarity matrix for amino acid sequences between proteins belonging to the nine-gene cluster. With the exception of RHPLAN_16830, which shared highest identity with RHPLAN_10480 (53.1 %), the remaining shared similarities were below 50 %. (b) Representation of highest identity for individual proteins in the cluster through blastp searches against 2323 bacterial genomes. The arrows represent the highest shared identity for each protein. Of the nine proteins in the array, only three members shared highest amino acid identity with other members of the same array, while four members shared highest identity with RHPLAN_29570, located elsewhere in the same genome. Two members were more similar to the TTT SBP ANW05692.1_3832 from *Bradyrhizobium icense* than to any gene inside the genome of *Rhodoplanes* sp. Z2-YC6860.

Within the unusual nine-gene array in *Rhodoplanes*, we examined the relationships between the proteins encoded by genes in the cluster (Fig. 3). Interestingly, with a single exception, the proteins are not more than 50 % identical to each other (Fig. 3a) and five of the nine proteins have the greatest identity to proteins outside the cluster, with RHPLAN_29570 being the closest match for four of them and the protein encoded by the gene ANW05692.1_3832 from *Bradyrhizobium icense* being the closest match for another two (Fig. 3b). Although an inverse correlation between cluster size and protein similarity has been previously observed in other studies [7], even this limited analysis indicates a complex pattern of gene gain and movement of genes around the chromosome.

Finally, we considered what the physiological role of this expansion might be and noted that it appears to have occurred multiple times in different bacteria. From the genome context of the TTT SBPs we could see that many were genetically linked to known transporters. However, only nine form part of standard operons for a full TTT transporter, along with genes encoding TctA and TctB-like membrane proteins. Perhaps more surprisingly, some are linked to genes encoding subunits of *other* SBP-dependent transporters, with eight linked to tripartite ATP-independent periplasmic (TRAP) transporters and 41 to ATP-binding cassette (ABC) transporters. One of the proposed explanations for the over-representation of TTT SBPs in members of the genus *Bordetella* was that these SBPs would interact with different TTT systems, and that one membrane component would be capable of interacting with different SBPs under diverse circumstances, which could also be the case in members of the genus *Rhodoplanes* [16, 23]. Another possibility would be that these orphan proteins are required for processes other than transport, where the binding protein function is related to chemical sensing and signalling rather than to transport directly, in systems such as two-component systems and the chemosensory apparatus that regulates chemotaxis. For example, this sensory role has been demonstrated for the BctDECBA system of *Bordetella pertussis* [28, 29]. Our searches in members of the genus *Rhodoplanes* revealed that 14 out of the 22 identified histidine kinase genes, as well as five genes encoding proteins related to flagella motility had genes encoding TTT SBPs nearby. In addition, 70 *tctC* genes (16 %) had a transcription factor encoded in their immediate vicinity. The abundance of transcription factor genes around TTT SBP genes has been previously observed in the genome of *Ralstonia eutropha*, where 64.1 % of the 154 homologs exhibited this characteristic [30]. From the 434 TctC sequences in *Rhodoplanes* sp., 355 (82 %) were predicted to have a signal for periplasmic export, identified by SignalP software [31], showing that regardless of their function, they are likely to be located in the periplasm, as expected.

The proteobacterial groups we have observed to show a TTT SBP expansion all inhabit complex soil and water environments, with the exception of some species of the genus *Bordetella*, known for their pathogenicity, but it has been speculated whether in the latter case this over-representation was inherited from a free-living ancestor and that these genes are gradually being lost [16]. The genus *Rhodoplanes* was first proposed by Hiraishi and Ueda [32] to comprise pink-coloured non-sulphur purple bacteria, closely related to the members of the genus *Rhodopseudomonas*, but forming an exclusive cluster in 16S rRNA phylogenetic analyses. Inhabiting a wide range of complex soil and water environments, they are known to have a diverse and versatile metabolism [33–36]. We speculate that this vast array of TTT SBPs could increase this versatility by providing an evolutionary advantage in terms of sensing, response and uptake in a complex environment in a more tailored fashion [7]. Following our genomic analyses, further transcriptomic and proteomic studies would be crucial in determining how physiologically relevant these proteins are. The polyphyletic origins of TTT expansion support the hypothesis that LSE played a major role in the mechanism of the expansion, potentially following ancient small events of HGT, indicating that environmental pressure can independently drive SBP duplication and adaptation from multiple starting points given the right environmental conditions.

### Conclusion

The present study describes a vast over-representation of TTT SBPs in the genome of the soil bacterium *Rhodoplanes* sp. Z2-YC6860, with 434 representatives. Although we cannot totally exclude the possibility that this gene family over-representation was partially driven by HGT, our findings indicate a major role of LSE in the origin of such an astonishing number of homologous genes, which may be linked to an increased metabolic versatility in this organism. Furthermore, we speculate that environmental pressure can drive SBP proliferation from multiple starting points. To the best of our knowledge, this is the most abundant gene family so far described in a prokaryotic genome.

## Supplementary Data

1641 Supplementary File 1
