# Supplementary material for: Massive over-representation of solute-binding proteins (SBPs) from the tripartite tricarboxylate transporter (TTT) family in the genome of the α-proteobacterium Rhodoplanes sp. Z2-YC6860
Source: Microb Genom. 2018 Apr 18;4(5):e000176. doi: 10.1099/mgen.0.000176 (PMC5994714; doi:10.1099/mgen.0.000176)
Supplement: Supplementary File 1 [file mgen-4-176-s001.pdf]

Suplementary Table 1 for the manuscript "Massive overrepresentation of solute binding proteins (SBPs) from the tripartite tricarboxylate transporter
Suplementary Table 1: TBLASTN searches of the 434 TctC homologs from *Rhodoplanes sp.* Z2-YC6860 against the complete genomes of 2323 dif

		**Best blast**		
**Rhodoplanes sp Z2 query sequence ID**	**Rhodoplanes sp Z2 query sequence (locus tag)**	**Subclass**	**Species/Accession**	**Assembly accession**
AMN43374.1_4909	RHPLAN_49500	Alphaproteobacteria	Bosea sp PAMC 26642	GCA_001562255.1
AMN38755.1_290	RHPLAN_02900	Alphaproteobacteria	Bradyrhizobium diazoefficiens USDA 110	GCA_000011365.1
AMN42205.1_3740	RHPLAN_37730	Alphaproteobacteria	Bradyrhizobium diazoefficiens USDA 110	GCA_000011365.1
AMN44588.1_6123	RHPLAN_61770	Alphaproteobacteria	Bradyrhizobium icense	GCA_001693385.1
AMN40138.1_1673	RHPLAN_16830	Alphaproteobacteria	Bradyrhizobium icense	GCA_001693385.1
AMN40365.1_1900	RHPLAN_19140	Alphaproteobacteria	Bradyrhizobium icense	GCA_001693385.1
AMN42074.1_3609	RHPLAN_36420	Alphaproteobacteria	Bradyrhizobium icense	GCA_001693385.1
AMN42447.1_3982	RHPLAN_40150	Alphaproteobacteria	Bradyrhizobium icense	GCA_001693385.1
AMN39510.1_1045	RHPLAN_10480	Alphaproteobacteria	Bradyrhizobium icense	GCA_001693385.1
AMN40610.1_2145	RHPLAN_21700	Alphaproteobacteria	Bradyrhizobium icense	GCA_001693385.1
AMN39129.1_664	RHPLAN_06670	Alphaproteobacteria	Bradyrhizobium icense	GCA_001693385.1
AMN42029.1_3564	RHPLAN_35970	Alphaproteobacteria	Bradyrhizobium icense	GCA_001693385.1
AMN40139.1_1674	RHPLAN_16840	Alphaproteobacteria	Bradyrhizobium icense	GCA_001693385.1
AMN39236.1_771	RHPLAN_07740	Alphaproteobacteria	Bradyrhizobium icense	GCA_001693385.1
AMN44544.1_6079	RHPLAN_61330	Alphaproteobacteria	Bradyrhizobium icense	GCA_001693385.1
AMN41087.1_2622	RHPLAN_26490	Alphaproteobacteria	Bradyrhizobium icense	GCA_001693385.1
AMN42032.1_3567	RHPLAN_36000	Alphaproteobacteria	Bradyrhizobium icense	GCA_001693385.1
AMN44352.1_5887	RHPLAN_59410	Alphaproteobacteria	Bradyrhizobium icense	GCA_001693385.1
AMN41027.1_2562	RHPLAN_25890	Alphaproteobacteria	Bradyrhizobium japonicum USDA 6	GCA_000284375.1
AMN40110.1_1645	RHPLAN_16550	Alphaproteobacteria	Bradyrhizobium japonicum USDA 6	GCA_000284375.1
AMN40369.1_1904	RHPLAN_19180	Alphaproteobacteria	Bradyrhizobium japonicum USDA 6	GCA_000284375.1
AMN44534.1_6069	RHPLAN_61230	Alphaproteobacteria	Bradyrhizobium oligotrophicum S58	GCA_000344805.1
AMN45483.1_7018	RHPLAN_70780	Alphaproteobacteria	Bradyrhizobium oligotrophicum S58	GCA_000344805.1
AMN41035.1_2570	RHPLAN_25970	Alphaproteobacteria	Bradyrhizobium oligotrophicum S58	GCA_000344805.1
AMN44583.1_6118	RHPLAN_61720	Alphaproteobacteria	Bradyrhizobium oligotrophicum S58	GCA_000344805.1

AMN44756.1_6291	RHPLAN_63500	Alphaproteobacteria	Polymorphum gilvum SL003B 26A1	GCA_000192745.1
AMN39763.1_1298	RHPLAN_13080	Alphaproteobacteria	Pseudorhodoplanes sinuspersici	GCA_002119765.1
AMN42217.1_3752	RHPLAN_37850	Alphaproteobacteria	Pseudorhodoplanes sinuspersici	GCA_002119765.1
AMN39434.1_969	RHPLAN_09720	Alphaproteobacteria	Pseudorhodoplanes sinuspersici	GCA_002119765.1
AMN44166.1_5701	RHPLAN_57520	Alphaproteobacteria	Pseudorhodoplanes sinuspersici	GCA_002119765.1
AMN39632.1_1167	RHPLAN_11700	Alphaproteobacteria	Pseudorhodoplanes sinuspersici	GCA_002119765.1
AMN42955.1_4490	RHPLAN_45260	Alphaproteobacteria	Pseudorhodoplanes sinuspersici	GCA_002119765.1
AMN41213.1_2748	RHPLAN_27760	Alphaproteobacteria	Pseudorhodoplanes sinuspersici	GCA_002119765.1
AMN43240.1_4775	RHPLAN_48160	Alphaproteobacteria	Pseudorhodoplanes sinuspersici	GCA_002119765.1
AMN39776.1_1311	RHPLAN_13210	Alphaproteobacteria	Pseudorhodoplanes sinuspersici	GCA_002119765.1
AMN44322.1_5857	RHPLAN_59110	Alphaproteobacteria	Pseudorhodoplanes sinuspersici	GCA_002119765.1
AMN43305.1_4840	RHPLAN_48810	Alphaproteobacteria	Pseudorhodoplanes sinuspersici	GCA_002119765.1
AMN43332.1_4867	RHPLAN_49080	Alphaproteobacteria	Pseudorhodoplanes sinuspersici	GCA_002119765.1
AMN39508.1_1043	RHPLAN_10460	Alphaproteobacteria	Pseudorhodoplanes sinuspersici	GCA_002119765.1
AMN41662.1_3197	RHPLAN_32270	Alphaproteobacteria	Pseudorhodoplanes sinuspersici	GCA_002119765.1
AMN41993.1_3528	RHPLAN_35610	Alphaproteobacteria	Pseudorhodoplanes sinuspersici	GCA_002119765.1
AMN39010.1_545	RHPLAN_05450	Alphaproteobacteria	Pseudorhodoplanes sinuspersici	GCA_002119765.1
AMN41859.1_3394	RHPLAN_34250	Alphaproteobacteria	Pseudorhodoplanes sinuspersici	GCA_002119765.1
AMN42877.1_4412	RHPLAN_44480	Alphaproteobacteria	Pseudorhodoplanes sinuspersici	GCA_002119765.1
AMN38828.1_363	RHPLAN_03630	Alphaproteobacteria	Pseudorhodoplanes sinuspersici	GCA_002119765.1
AMN43245.1_4780	RHPLAN_48210	Alphaproteobacteria	Pseudorhodoplanes sinuspersici	GCA_002119765.1
AMN44973.1_6508	RHPLAN_65670	Alphaproteobacteria	Pseudorhodoplanes sinuspersici	GCA_002119765.1
AMN44356.1_5891	RHPLAN_59450	Alphaproteobacteria	Pseudorhodoplanes sinuspersici	GCA_002119765.1
AMN40249.1_1784	RHPLAN_17980	Alphaproteobacteria	Pseudorhodoplanes sinuspersici	GCA_002119765.1
AMN44487.1_6022	RHPLAN_60760	Alphaproteobacteria	Pseudorhodoplanes sinuspersici	GCA_002119765.1
AMN44204.1_5739	RHPLAN_57900	Alphaproteobacteria	Pseudorhodoplanes sinuspersici	GCA_002119765.1
AMN41445.1_2980	RHPLAN_30090	Alphaproteobacteria	Pseudorhodoplanes sinuspersici	GCA_002119765.1
AMN42300.1_3835	RHPLAN_38680	Alphaproteobacteria	Pseudorhodoplanes sinuspersici	GCA_002119765.1
AMN43007.1_4542	RHPLAN_45780	Alphaproteobacteria	Pseudorhodoplanes sinuspersici	GCA_002119765.1
AMN43070.1_4605	RHPLAN_46420	Alphaproteobacteria	Pseudorhodoplanes sinuspersici	GCA_002119765.1
AMN40250.1_1785	RHPLAN_17990	Alphaproteobacteria	Pseudorhodoplanes sinuspersici	GCA_002119765.1
AMN43042.1_4577	RHPLAN_46130	Alphaproteobacteria	Pseudorhodoplanes sinuspersici	GCA_002119765.1

AMN41454.1_2989	RHPLAN_30180	Alphaproteobacteria	Pseudorhodoplanes sinuspersici	GCA_002119765.1
AMN41675.1_3210	RHPLAN_32410	Alphaproteobacteria	Pseudorhodoplanes sinuspersici	GCA_002119765.1
AMN41379.1_2914	RHPLAN_29420	Alphaproteobacteria	Pseudorhodoplanes sinuspersici	GCA_002119765.1
AMN41856.1_3391	RHPLAN_34220	Alphaproteobacteria	Pseudorhodoplanes sinuspersici	GCA_002119765.1
AMN40639.1_2174	RHPLAN_21990	Alphaproteobacteria	Pseudorhodoplanes sinuspersici	GCA_002119765.1
AMN44205.1_5740	RHPLAN_57910	Alphaproteobacteria	Pseudorhodoplanes sinuspersici	GCA_002119765.1
AMN42765.1_4300	RHPLAN_43360	Alphaproteobacteria	Pseudorhodoplanes sinuspersici	GCA_002119765.1
AMN40938.1_2473	RHPLAN_25000	Alphaproteobacteria	Pseudorhodoplanes sinuspersici	GCA_002119765.1
AMN40248.1_1783	RHPLAN_17970	Alphaproteobacteria	Pseudorhodoplanes sinuspersici	GCA_002119765.1
AMN40290.1_1825	RHPLAN_18390	Alphaproteobacteria	Pseudorhodoplanes sinuspersici	GCA_002119765.1
AMN39471.1_1006	RHPLAN_10090	Alphaproteobacteria	Pseudorhodoplanes sinuspersici	GCA_002119765.1
AMN41270.1_2805	RHPLAN_28330	Alphaproteobacteria	Pseudorhodoplanes sinuspersici	GCA_002119765.1
AMN40792.1_2327	RHPLAN_23520	Alphaproteobacteria	Pseudorhodoplanes sinuspersici	GCA_002119765.1
AMN42662.1_4197	RHPLAN_42310	Alphaproteobacteria	Pseudorhodoplanes sinuspersici	GCA_002119765.1
AMN45209.1_6744	RHPLAN_68030	Alphaproteobacteria	Pseudorhodoplanes sinuspersici	GCA_002119765.1
AMN44591.1_6126	RHPLAN_61800	Alphaproteobacteria	Pseudorhodoplanes sinuspersici	GCA_002119765.1
AMN39917.1_1452	RHPLAN_14620	Alphaproteobacteria	Pseudorhodoplanes sinuspersici	GCA_002119765.1
AMN39598.1_1133	RHPLAN_11360	Alphaproteobacteria	Pseudorhodoplanes sinuspersici	GCA_002119765.1
AMN41459.1_2994	RHPLAN_30230	Alphaproteobacteria	Pseudorhodoplanes sinuspersici	GCA_002119765.1
AMN39470.1_1005	RHPLAN_10080	Alphaproteobacteria	Pseudorhodoplanes sinuspersici	GCA_002119765.1
AMN40000.1_1535	RHPLAN_15450	Alphaproteobacteria	Pseudorhodoplanes sinuspersici	GCA_002119765.1
AMN44266.1_5801	RHPLAN_58540	Alphaproteobacteria	Pseudorhodoplanes sinuspersici	GCA_002119765.1
AMN39835.1_1370	RHPLAN_13800	Alphaproteobacteria	Pseudorhodoplanes sinuspersici	GCA_002119765.1
AMN39468.1_1003	RHPLAN_10060	Alphaproteobacteria	Pseudorhodoplanes sinuspersici	GCA_002119765.1
AMN40068.1_1603	RHPLAN_16130	Alphaproteobacteria	Pseudorhodoplanes sinuspersici	GCA_002119765.1
AMN42139.1_3674	RHPLAN_37070	Alphaproteobacteria	Pseudorhodoplanes sinuspersici	GCA_002119765.1
AMN39515.1_1050	RHPLAN_10530	Alphaproteobacteria	Pseudorhodoplanes sinuspersici	GCA_002119765.1
AMN40964.1_2499	RHPLAN_25260	Alphaproteobacteria	Pseudorhodoplanes sinuspersici	GCA_002119765.1
AMN39302.1_837	RHPLAN_08400	Alphaproteobacteria	Pseudorhodoplanes sinuspersici	GCA_002119765.1
AMN39348.1_883	RHPLAN_08860	Alphaproteobacteria	Pseudorhodoplanes sinuspersici	GCA_002119765.1
AMN43150.1_4685	RHPLAN_47240	Alphaproteobacteria	Pseudorhodoplanes sinuspersici	GCA_002119765.1
AMN39448.1_983	RHPLAN_09860	Alphaproteobacteria	Pseudorhodoplanes sinuspersici	GCA_002119765.1

AMN40101.1_1636	RHPLAN_16460	Alphaproteobacteria	Pseudorhodoplanes sinuspersici	GCA_002119765.1
AMN43187.1_4722	RHPLAN_47630	Alphaproteobacteria	Pseudorhodoplanes sinuspersici	GCA_002119765.1
AMN44949.1_6484	RHPLAN_65430	Alphaproteobacteria	Pseudorhodoplanes sinuspersici	GCA_002119765.1
AMN45221.1_6756	RHPLAN_68150	Alphaproteobacteria	Pseudorhodoplanes sinuspersici	GCA_002119765.1
AMN42216.1_3751	RHPLAN_37840	Alphaproteobacteria	Pseudorhodoplanes sinuspersici	GCA_002119765.1
AMN41777.1_3312	RHPLAN_33430	Alphaproteobacteria	Pseudorhodoplanes sinuspersici	GCA_002119765.1
AMN45337.1_6872	RHPLAN_69310	Alphaproteobacteria	Pseudorhodoplanes sinuspersici	GCA_002119765.1
AMN43556.1_5091	RHPLAN_51330	Alphaproteobacteria	Pseudorhodoplanes sinuspersici	GCA_002119765.1
AMN39285.1_820	RHPLAN_08230	Alphaproteobacteria	Pseudorhodoplanes sinuspersici	GCA_002119765.1
AMN44660.1_6195	RHPLAN_62490	Alphaproteobacteria	Rhodoplanes sp Z2 YC6860	GCA_001579845.1
AMN41105.1_2640	RHPLAN_26680	Alphaproteobacteria	Rhodoplanes sp Z2 YC6860	GCA_001579845.1
AMN44679.1_6214	RHPLAN_62680	Alphaproteobacteria	Rhodoplanes sp Z2 YC6860	GCA_001579845.1
AMN44853.1_6388	RHPLAN_64470	Alphaproteobacteria	Rhodoplanes sp Z2 YC6860	GCA_001579845.1
AMN45417.1_6952	RHPLAN_70110	Alphaproteobacteria	Rhodoplanes sp Z2 YC6860	GCA_001579845.1
AMN45418.1_6953	RHPLAN_70120	Alphaproteobacteria	Rhodoplanes sp Z2 YC6860	GCA_001579845.1
AMN45497.1_7032	RHPLAN_70920	Alphaproteobacteria	Rhodoplanes sp Z2 YC6860	GCA_001579845.1
AMN45498.1_7033	RHPLAN_70930	Alphaproteobacteria	Rhodoplanes sp Z2 YC6860	GCA_001579845.1
AMN43565.1_5100	RHPLAN_51420	Alphaproteobacteria	Rhodoplanes sp Z2 YC6860	GCA_001579845.1
AMN39232.1_767	RHPLAN_07700	Alphaproteobacteria	Rhodoplanes sp Z2 YC6860	GCA_001579845.1
AMN42088.1_3623	RHPLAN_36560	Alphaproteobacteria	Rhodoplanes sp Z2 YC6860	GCA_001579845.1
AMN39081.1_616	RHPLAN_06190	Alphaproteobacteria	Rhodoplanes sp Z2 YC6860	GCA_001579845.1
AMN43897.1_5432	RHPLAN_54810	Alphaproteobacteria	Rhodoplanes sp Z2 YC6860	GCA_001579845.1
AMN44369.1_5904	RHPLAN_59580	Alphaproteobacteria	Rhodoplanes sp Z2 YC6860	GCA_001579845.1
AMN44387.1_5922	RHPLAN_59760	Alphaproteobacteria	Rhodoplanes sp Z2 YC6860	GCA_001579845.1
AMN44552.1_6087	RHPLAN_61410	Alphaproteobacteria	Rhodoplanes sp Z2 YC6860	GCA_001579845.1
AMN43264.1_4799	RHPLAN_48400	Alphaproteobacteria	Rhodoplanes sp Z2 YC6860	GCA_001579845.1
AMN41292.1_2827	RHPLAN_28550	Alphaproteobacteria	Rhodoplanes sp Z2 YC6860	GCA_001579845.1
AMN42620.1_4155	RHPLAN_41890	Alphaproteobacteria	Rhodoplanes sp Z2 YC6860	GCA_001579845.1
AMN39299.1_834	RHPLAN_08370	Alphaproteobacteria	Rhodoplanes sp Z2 YC6860	GCA_001579845.1
AMN41457.1_2992	RHPLAN_30210	Alphaproteobacteria	Rhodoplanes sp Z2 YC6860	GCA_001579845.1
AMN42292.1_3827	RHPLAN_38600	Alphaproteobacteria	Rhodoplanes sp Z2 YC6860	GCA_001579845.1
AMN39298.1_833	RHPLAN_08360	Alphaproteobacteria	Rhodoplanes sp Z2 YC6860	GCA_001579845.1

AMN42083.1_3618	RHPLAN_36510	Alphaproteobacteria	Rhodoplanes sp Z2 YC6860	GCA_001579845.1
AMN42082.1_3617	RHPLAN_36500	Alphaproteobacteria	Rhodoplanes sp Z2 YC6860	GCA_001579845.1
AMN39858.1_1393	RHPLAN_14030	Alphaproteobacteria	Rhodoplanes sp Z2 YC6860	GCA_001579845.1
AMN41822.1_3357	RHPLAN_33880	Alphaproteobacteria	Rhodoplanes sp Z2 YC6860	GCA_001579845.1
AMN42802.1_4337	RHPLAN_43730	Alphaproteobacteria	Rhodoplanes sp Z2 YC6860	GCA_001579845.1
AMN39857.1_1392	RHPLAN_14020	Alphaproteobacteria	Rhodoplanes sp Z2 YC6860	GCA_001579845.1
AMN39623.1_1158	RHPLAN_11610	Alphaproteobacteria	Rhodoplanes sp Z2 YC6860	GCA_001579845.1
AMN41455.1_2990	RHPLAN_30190	Alphaproteobacteria	Rhodoplanes sp Z2 YC6860	GCA_001579845.1
AMN43852.1_5387	RHPLAN_54360	Alphaproteobacteria	Rhodoplanes sp Z2 YC6860	GCA_001579845.1
AMN44966.1_6501	RHPLAN_65600	Alphaproteobacteria	Rhodoplanes sp Z2 YC6860	GCA_001579845.1
AMN45491.1_7026	RHPLAN_70860	Alphaproteobacteria	Rhodoplanes sp Z2 YC6860	GCA_001579845.1
AMN40380.1_1915	RHPLAN_19290	Alphaproteobacteria	Rhodoplanes sp Z2 YC6860	GCA_001579845.1
AMN40814.1_2349	RHPLAN_23740	Alphaproteobacteria	Rhodoplanes sp Z2 YC6860	GCA_001579845.1
AMN39337.1_872	RHPLAN_08750	Alphaproteobacteria	Rhodoplanes sp Z2 YC6860	GCA_001579845.1
AMN42826.1_4361	RHPLAN_43970	Alphaproteobacteria	Rhodoplanes sp Z2 YC6860	GCA_001579845.1
AMN45310.1_6845	RHPLAN_69040	Alphaproteobacteria	Rhodoplanes sp Z2 YC6860	GCA_001579845.1
AMN41190.1_2725	RHPLAN_27530	Alphaproteobacteria	Rhodoplanes sp Z2 YC6860	GCA_001579845.1
AMN41885.1_3420	RHPLAN_34510	Alphaproteobacteria	Rhodoplanes sp Z2 YC6860	GCA_001579845.1
AMN42411.1_3946	RHPLAN_39790	Alphaproteobacteria	Rhodoplanes sp Z2 YC6860	GCA_001579845.1
AMN41396.1_2931	RHPLAN_29590	Alphaproteobacteria	Rhodoplanes sp Z2 YC6860	GCA_001579845.1
AMN39497.1_1032	RHPLAN_10350	Alphaproteobacteria	Rhodoplanes sp Z2 YC6860	GCA_001579845.1
AMN41130.1_2665	RHPLAN_26930	Alphaproteobacteria	Rhodoplanes sp Z2 YC6860	GCA_001579845.1
AMN39809.1_1344	RHPLAN_13540	Alphaproteobacteria	Rhodoplanes sp Z2 YC6860	GCA_001579845.1
AMN40100.1_1635	RHPLAN_16450	Alphaproteobacteria	Rhodoplanes sp Z2 YC6860	GCA_001579845.1
AMN41488.1_3023	RHPLAN_30520	Alphaproteobacteria	Rhodoplanes sp Z2 YC6860	GCA_001579845.1
AMN39888.1_1423	RHPLAN_14330	Alphaproteobacteria	Rhodoplanes sp Z2 YC6860	GCA_001579845.1
AMN40140.1_1675	RHPLAN_16850	Alphaproteobacteria	Rhodoplanes sp Z2 YC6860	GCA_001579845.1
AMN41398.1_2933	RHPLAN_29610	Alphaproteobacteria	Rhodoplanes sp Z2 YC6860	GCA_001579845.1
AMN38592.1_127	RHPLAN_01270	Alphaproteobacteria	Rhodoplanes sp Z2 YC6860	GCA_001579845.1
AMN39300.1_835	RHPLAN_08380	Alphaproteobacteria	Rhodoplanes sp Z2 YC6860	GCA_001579845.1
AMN43389.1_4924	RHPLAN_49650	Alphaproteobacteria	Rhodoplanes sp Z2 YC6860	GCA_001579845.1
AMN44921.1_6456	RHPLAN_65150	Alphaproteobacteria	Rhodoplanes sp Z2 YC6860	GCA_001579845.1

AMN43185.1_4720	RHPLAN_47610	Alphaproteobacteria	Rhodoplanes sp Z2 YC6860	GCA_001579845.1
AMN39494.1_1029	RHPLAN_10320	Alphaproteobacteria	Rhodoplanes sp Z2 YC6860	GCA_001579845.1
AMN41229.1_2764	RHPLAN_27920	Alphaproteobacteria	Rhodoplanes sp Z2 YC6860	GCA_001579845.1
AMN41644.1_3179	RHPLAN_32090	Alphaproteobacteria	Rhodoplanes sp Z2 YC6860	GCA_001579845.1
AMN41586.1_3121	RHPLAN_31510	Alphaproteobacteria	Rhodoplanes sp Z2 YC6860	GCA_001579845.1
AMN40364.1_1899	RHPLAN_19130	Alphaproteobacteria	Rhodoplanes sp Z2 YC6860	GCA_001579845.1
AMN39037.1_572	RHPLAN_05720	Alphaproteobacteria	Rhodoplanes sp Z2 YC6860	GCA_001579845.1
AMN40540.1_2075	RHPLAN_21000	Alphaproteobacteria	Rhodoplanes sp Z2 YC6860	GCA_001579845.1
AMN38594.1_129	RHPLAN_01290	Alphaproteobacteria	Rhodoplanes sp Z2 YC6860	GCA_001579845.1
AMN42361.1_3896	RHPLAN_39290	Alphaproteobacteria	Rhodoplanes sp Z2 YC6860	GCA_001579845.1
AMN43237.1_4772	RHPLAN_48130	Alphaproteobacteria	Rhodoplanes sp Z2 YC6860	GCA_001579845.1
AMN44922.1_6457	RHPLAN_65160	Alphaproteobacteria	Rhodoplanes sp Z2 YC6860	GCA_001579845.1
AMN41041.1_2576	RHPLAN_26030	Alphaproteobacteria	Rhodoplanes sp Z2 YC6860	GCA_001579845.1
AMN41394.1_2929	RHPLAN_29570	Alphaproteobacteria	Rhodoplanes sp Z2 YC6860	GCA_001579845.1
AMN43390.1_4925	RHPLAN_49660	Alphaproteobacteria	Rhodoplanes sp Z2 YC6860	GCA_001579845.1
AMN41773.1_3308	RHPLAN_33390	Alphaproteobacteria	Rhodoplanes sp Z2 YC6860	GCA_001579845.1
AMN40300.1_1835	RHPLAN_18490	Alphaproteobacteria	Rhodoplanes sp Z2 YC6860	GCA_001579845.1
AMN40384.1_1919	RHPLAN_19330	Alphaproteobacteria	Rhodoplanes sp Z2 YC6860	GCA_001579845.1
AMN39430.1_965	RHPLAN_09680	Alphaproteobacteria	Rhodoplanes sp Z2 YC6860	GCA_001579845.1
AMN40864.1_2399	RHPLAN_24240	Alphaproteobacteria	Rhodoplanes sp Z2 YC6860	GCA_001579845.1
AMN42630.1_4165	RHPLAN_41990	Alphaproteobacteria	Rhodoplanes sp Z2 YC6860	GCA_001579845.1
AMN38593.1_128	RHPLAN_01280	Alphaproteobacteria	Rhodoplanes sp Z2 YC6860	GCA_001579845.1
AMN43839.1_5374	RHPLAN_54230	Alphaproteobacteria	Rhodoplanes sp Z2 YC6860	GCA_001579845.1
AMN41325.1_2860	RHPLAN_28880	Alphaproteobacteria	Rhodoplanes sp Z2 YC6860	GCA_001579845.1
AMN43276.1_4811	RHPLAN_48520	Alphaproteobacteria	Rhodoplanes sp Z2 YC6860	GCA_001579845.1
AMN43810.1_5345	RHPLAN_53940	Alphaproteobacteria	Rhodoplanes sp Z2 YC6860	GCA_001579845.1
AMN40761.1_2296	RHPLAN_23210	Alphaproteobacteria	Rhodoplanes sp Z2 YC6860	GCA_001579845.1
AMN44346.1_5881	RHPLAN_59350	Alphaproteobacteria	Rhodoplanes sp Z2 YC6860	GCA_001579845.1
AMN40791.1_2326	RHPLAN_23510	Alphaproteobacteria	Rhodoplanes sp Z2 YC6860	GCA_001579845.1
AMN40114.1_1649	RHPLAN_16590	Alphaproteobacteria	Rhodoplanes sp Z2 YC6860	GCA_001579845.1
AMN41040.1_2575	RHPLAN_26020	Alphaproteobacteria	Rhodoplanes sp Z2 YC6860	GCA_001579845.1
AMN42545.1_4080	RHPLAN_41140	Alphaproteobacteria	Rhodoplanes sp Z2 YC6860	GCA_001579845.1

AMN41827.1_3362	RHPLAN_33930	Alphaproteobacteria	Rhodoplanes sp Z2 YC6860	GCA_001579845.1
AMN43514.1_5049	RHPLAN_50910	Alphaproteobacteria	Rhodoplanes sp Z2 YC6860	GCA_001579845.1
AMN39836.1_1371	RHPLAN_13810	Alphaproteobacteria	Rhodoplanes sp Z2 YC6860	GCA_001579845.1
AMN40809.1_2344	RHPLAN_23690	Alphaproteobacteria	Rhodoplanes sp Z2 YC6860	GCA_001579845.1
AMN38699.1_234	RHPLAN_02340	Alphaproteobacteria	Rhodoplanes sp Z2 YC6860	GCA_001579845.1
AMN40385.1_1920	RHPLAN_19340	Alphaproteobacteria	Rhodoplanes sp Z2 YC6860	GCA_001579845.1
AMN42861.1_4396	RHPLAN_44320	Alphaproteobacteria	Rhodoplanes sp Z2 YC6860	GCA_001579845.1
AMN44952.1_6487	RHPLAN_65460	Alphaproteobacteria	Rhodoplanes sp Z2 YC6860	GCA_001579845.1
AMN42703.1_4238	RHPLAN_42730	Alphaproteobacteria	Rhodoplanes sp Z2 YC6860	GCA_001579845.1
AMN41267.1_2802	RHPLAN_28300	Alphaproteobacteria	Rhodoplanes sp Z2 YC6860	GCA_001579845.1
AMN44496.1_6031	RHPLAN_60850	Alphaproteobacteria	Rhodoplanes sp Z2 YC6860	GCA_001579845.1
AMN45416.1_6951	RHPLAN_70100	Alphaproteobacteria	Rhodoplanes sp Z2 YC6860	GCA_001579845.1
AMN41517.1_3052	RHPLAN_30810	Alphaproteobacteria	Rhodoplanes sp Z2 YC6860	GCA_001579845.1
AMN43568.1_5103	RHPLAN_51450	Alphaproteobacteria	Rhodoplanes sp Z2 YC6860	GCA_001579845.1
AMN38644.1_179	RHPLAN_01790	Alphaproteobacteria	Rhodoplanes sp Z2 YC6860	GCA_001579845.1
AMN44072.1_5607	RHPLAN_56560	Alphaproteobacteria	Rhodoplanes sp Z2 YC6860	GCA_001579845.1
AMN39306.1_841	RHPLAN_08440	Alphaproteobacteria	Rhodoplanes sp Z2 YC6860	GCA_001579845.1
AMN41728.1_3263	RHPLAN_32940	Alphaproteobacteria	Rhodoplanes sp Z2 YC6860	GCA_001579845.1
AMN44609.1_6144	RHPLAN_61980	Alphaproteobacteria	Rhodoplanes sp Z2 YC6860	GCA_001579845.1
AMN45390.1_6925	RHPLAN_69840	Alphaproteobacteria	Rhodoplanes sp Z2 YC6860	GCA_001579845.1
AMN42478.1_4013	RHPLAN_40460	Alphaproteobacteria	Rhodoplanes sp Z2 YC6860	GCA_001579845.1
AMN39308.1_843	RHPLAN_08460	Alphaproteobacteria	Rhodoplanes sp Z2 YC6860	GCA_001579845.1
AMN39597.1_1132	RHPLAN_11350	Alphaproteobacteria	Rhodoplanes sp Z2 YC6860	GCA_001579845.1
AMN40141.1_1676	RHPLAN_16860	Alphaproteobacteria	Rhodoplanes sp Z2 YC6860	GCA_001579845.1
AMN41768.1_3303	RHPLAN_33340	Alphaproteobacteria	Rhodoplanes sp Z2 YC6860	GCA_001579845.1
AMN39418.1_953	RHPLAN_09560	Alphaproteobacteria	Rhodoplanes sp Z2 YC6860	GCA_001579845.1
AMN42783.1_4318	RHPLAN_43540	Alphaproteobacteria	Rhodoplanes sp Z2 YC6860	GCA_001579845.1
AMN44046.1_5581	RHPLAN_56300	Alphaproteobacteria	Rhodoplanes sp Z2 YC6860	GCA_001579845.1
AMN40597.1_2132	RHPLAN_21570	Alphaproteobacteria	Rhodoplanes sp Z2 YC6860	GCA_001579845.1
AMN40891.1_2426	RHPLAN_24530	Alphaproteobacteria	Rhodoplanes sp Z2 YC6860	GCA_001579845.1
AMN41039.1_2574	RHPLAN_26010	Alphaproteobacteria	Rhodoplanes sp Z2 YC6860	GCA_001579845.1
AMN43364.1_4899	RHPLAN_49400	Alphaproteobacteria	Rhodoplanes sp Z2 YC6860	GCA_001579845.1

AMN39036.1_571	RHPLAN_05710	Alphaproteobacteria	Rhodoplanes sp Z2 YC6860	GCA_001579845.1
AMN43183.1_4718	RHPLAN_47590	Alphaproteobacteria	Rhodoplanes sp Z2 YC6860	GCA_001579845.1
AMN41860.1_3395	RHPLAN_34260	Alphaproteobacteria	Rhodoplanes sp Z2 YC6860	GCA_001579845.1
AMN39007.1_542	RHPLAN_05420	Alphaproteobacteria	Rhodoplanes sp Z2 YC6860	GCA_001579845.1
AMN45225.1_6760	RHPLAN_68190	Alphaproteobacteria	Rhodoplanes sp Z2 YC6860	GCA_001579845.1
AMN40137.1_1672	RHPLAN_16820	Alphaproteobacteria	Rhodoplanes sp Z2 YC6860	GCA_001579845.1
AMN39592.1_1127	RHPLAN_11300	Alphaproteobacteria	Rhodoplanes sp Z2 YC6860	GCA_001579845.1
AMN39966.1_1501	RHPLAN_15110	Alphaproteobacteria	Rhodoplanes sp Z2 YC6860	GCA_001579845.1
AMN42147.1_3682	RHPLAN_37150	Alphaproteobacteria	Rhodoplanes sp Z2 YC6860	GCA_001579845.1
AMN42450.1_3985	RHPLAN_40180	Alphaproteobacteria	Rhodoplanes sp Z2 YC6860	GCA_001579845.1
AMN43567.1_5102	RHPLAN_51440	Alphaproteobacteria	Rhodoplanes sp Z2 YC6860	GCA_001579845.1
AMN39534.1_1069	RHPLAN_10720	Alphaproteobacteria	Rhodoplanes sp Z2 YC6860	GCA_001579845.1
AMN43536.1_5071	RHPLAN_51130	Alphaproteobacteria	Rhodoplanes sp Z2 YC6860	GCA_001579845.1
AMN44737.1_6272	RHPLAN_63280	Alphaproteobacteria	Rhodoplanes sp Z2 YC6860	GCA_001579845.1
AMN43801.1_5336	RHPLAN_53850	Alphaproteobacteria	Rhodoplanes sp Z2 YC6860	GCA_001579845.1
AMN39243.1_778	RHPLAN_07810	Alphaproteobacteria	Rhodoplanes sp Z2 YC6860	GCA_001579845.1
AMN42646.1_4181	RHPLAN_42150	Alphaproteobacteria	Rhodoplanes sp Z2 YC6860	GCA_001579845.1
AMN41061.1_2596	RHPLAN_26230	Alphaproteobacteria	Rhodoplanes sp Z2 YC6860	GCA_001579845.1
AMN43546.1_5081	RHPLAN_51230	Alphaproteobacteria	Rhodoplanes sp Z2 YC6860	GCA_001579845.1
AMN41593.1_3128	RHPLAN_31580	Alphaproteobacteria	Rhodoplanes sp Z2 YC6860	GCA_001579845.1
AMN40935.1_2470	RHPLAN_24970	Alphaproteobacteria	Rhodoplanes sp Z2 YC6860	GCA_001579845.1
AMN44623.1_6158	RHPLAN_62120	Alphaproteobacteria	Rhodoplanes sp Z2 YC6860	GCA_001579845.1
AMN38591.1_126	RHPLAN_01260	Alphaproteobacteria	Rhodoplanes sp Z2 YC6860	GCA_001579845.1
AMN44225.1_5760	RHPLAN_58110	Alphaproteobacteria	Rhodoplanes sp Z2 YC6860	GCA_001579845.1
AMN40360.1_1895	RHPLAN_19090	Alphaproteobacteria	Rhodoplanes sp Z2 YC6860	GCA_001579845.1
AMN44910.1_6445	RHPLAN_65040	Alphaproteobacteria	Rhodoplanes sp Z2 YC6860	GCA_001579845.1
AMN42333.1_3868	RHPLAN_39010	Alphaproteobacteria	Rhodoplanes sp Z2 YC6860	GCA_001579845.1
AMN42155.1_3690	RHPLAN_37230	Alphaproteobacteria	Rhodoplanes sp Z2 YC6860	GCA_001579845.1
AMN44909.1_6444	RHPLAN_65030	Alphaproteobacteria	Rhodoplanes sp Z2 YC6860	GCA_001579845.1
AMN42618.1_4153	RHPLAN_41870	Alphaproteobacteria	Rhodoplanes sp Z2 YC6860	GCA_001579845.1
AMN43823.1_5358	RHPLAN_54070	Alphaproteobacteria	Rhodoplanes sp Z2 YC6860	GCA_001579845.1
AMN42332.1_3867	RHPLAN_39000	Alphaproteobacteria	Rhodoplanes sp Z2 YC6860	GCA_001579845.1

AMN42827.1_4362	RHPLAN_43980	Alphaproteobacteria	Rhodoplanes sp Z2 YC6860	GCA_001579845.1
AMN44001.1_5536	RHPLAN_55850	Alphaproteobacteria	Rhodoplanes sp Z2 YC6860	GCA_001579845.1
AMN42424.1_3959	RHPLAN_39920	Alphaproteobacteria	Rhodoplanes sp Z2 YC6860	GCA_001579845.1
AMN39560.1_1095	RHPLAN_10980	Alphaproteobacteria	Rhodoplanes sp Z2 YC6860	GCA_001579845.1
AMN41595.1_3130	RHPLAN_31600	Alphaproteobacteria	Rhodoplanes sp Z2 YC6860	GCA_001579845.1
AMN39231.1_766	RHPLAN_07690	Alphaproteobacteria	Rhodoplanes sp Z2 YC6860	GCA_001579845.1
AMN39999.1_1534	RHPLAN_15440	Alphaproteobacteria	Rhodoplanes sp Z2 YC6860	GCA_001579845.1
AMN43096.1_4631	RHPLAN_46680	Alphaproteobacteria	Rhodoplanes sp Z2 YC6860	GCA_001579845.1
AMN43246.1_4781	RHPLAN_48220	Alphaproteobacteria	Rhodoplanes sp Z2 YC6860	GCA_001579845.1
AMN43248.1_4783	RHPLAN_48240	Alphaproteobacteria	Rhodoplanes sp Z2 YC6860	GCA_001579845.1
AMN43371.1_4906	RHPLAN_49470	Alphaproteobacteria	Rhodoplanes sp Z2 YC6860	GCA_001579845.1
AMN40142.1_1677	RHPLAN_16870	Alphaproteobacteria	Rhodoplanes sp Z2 YC6860	GCA_001579845.1
AMN42841.1_4376	RHPLAN_44120	Alphaproteobacteria	Rhodoplanes sp Z2 YC6860	GCA_001579845.1
AMN44283.1_5818	RHPLAN_58720	Alphaproteobacteria	Rhodoplanes sp Z2 YC6860	GCA_001579845.1
AMN41760.1_3295	RHPLAN_33260	Alphaproteobacteria	Rhodoplanes sp Z2 YC6860	GCA_001579845.1
AMN42917.1_4452	RHPLAN_44880	Alphaproteobacteria	Rhodoplanes sp Z2 YC6860	GCA_001579845.1
AMN41211.1_2746	RHPLAN_27740	Alphaproteobacteria	Rhodoplanes sp Z2 YC6860	GCA_001579845.1
AMN44891.1_6426	RHPLAN_64850	Alphaproteobacteria	Rhodoplanes sp Z2 YC6860	GCA_001579845.1
AMN38788.1_323	RHPLAN_03230	Alphaproteobacteria	Rhodoplanes sp Z2 YC6860	GCA_001579845.1
AMN41858.1_3393	RHPLAN_34240	Alphaproteobacteria	Rhodoplanes sp Z2 YC6860	GCA_001579845.1
AMN39038.1_573	RHPLAN_05730	Alphaproteobacteria	Rhodoplanes sp Z2 YC6860	GCA_001579845.1
AMN40387.1_1922	RHPLAN_19360	Alphaproteobacteria	Rhodoplanes sp Z2 YC6860	GCA_001579845.1
AMN43841.1_5376	RHPLAN_54250	Alphaproteobacteria	Rhodoplanes sp Z2 YC6860	GCA_001579845.1
AMN39283.1_818	RHPLAN_08210	Alphaproteobacteria	Rhodoplanes sp Z2 YC6860	GCA_001579845.1
AMN44488.1_6023	RHPLAN_60770	Alphaproteobacteria	Rhodoplanes sp Z2 YC6860	GCA_001579845.1
AMN41935.1_3470	RHPLAN_35030	Alphaproteobacteria	Rhodoplanes sp Z2 YC6860	GCA_001579845.1
AMN39349.1_884	RHPLAN_08870	Alphaproteobacteria	Rhodoplanes sp Z2 YC6860	GCA_001579845.1
AMN39786.1_1321	RHPLAN_13310	Alphaproteobacteria	Rhodoplanes sp Z2 YC6860	GCA_001579845.1
AMN44555.1_6090	RHPLAN_61440	Alphaproteobacteria	Rhodoplanes sp Z2 YC6860	GCA_001579845.1
AMN40136.1_1671	RHPLAN_16810	Alphaproteobacteria	Rhodoplanes sp Z2 YC6860	GCA_001579845.1
AMN39039.1_574	RHPLAN_05740	Alphaproteobacteria	Rhodoplanes sp Z2 YC6860	GCA_001579845.1
AMN39533.1_1068	RHPLAN_10710	Alphaproteobacteria	Rhodoplanes sp Z2 YC6860	GCA_001579845.1

AMN41449.1_2984	RHPLAN_30130	Alphaproteobacteria	Rhodoplanes sp Z2 YC6860	GCA_001579845.1
AMN43577.1_5112	RHPLAN_51540	Alphaproteobacteria	Rhodoplanes sp Z2 YC6860	GCA_001579845.1
AMN41446.1_2981	RHPLAN_30100	Alphaproteobacteria	Rhodoplanes sp Z2 YC6860	GCA_001579845.1
AMN42350.1_3885	RHPLAN_39180	Alphaproteobacteria	Rhodoplanes sp Z2 YC6860	GCA_001579845.1
AMN44076.1_5611	RHPLAN_56600	Alphaproteobacteria	Rhodoplanes sp Z2 YC6860	GCA_001579845.1
AMN43969.1_5504	RHPLAN_55530	Alphaproteobacteria	Rhodoplanes sp Z2 YC6860	GCA_001579845.1
AMN41209.1_2744	RHPLAN_27720	Alphaproteobacteria	Rhodoplanes sp Z2 YC6860	GCA_001579845.1
AMN43825.1_5360	RHPLAN_54090	Alphaproteobacteria	Rhodoplanes sp Z2 YC6860	GCA_001579845.1
AMN41973.1_3508	RHPLAN_35410	Alphaproteobacteria	Rhodoplanes sp Z2 YC6860	GCA_001579845.1
AMN39621.1_1156	RHPLAN_11590	Alphaproteobacteria	Rhodoplanes sp Z2 YC6860	GCA_001579845.1
AMN44967.1_6502	RHPLAN_65610	Alphaproteobacteria	Rhodoplanes sp Z2 YC6860	GCA_001579845.1
AMN39872.1_1407	RHPLAN_14170	Alphaproteobacteria	Rhodoplanes sp Z2 YC6860	GCA_001579845.1
AMN40144.1_1679	RHPLAN_16890	Alphaproteobacteria	Rhodoplanes sp Z2 YC6860	GCA_001579845.1
AMN40388.1_1923	RHPLAN_19370	Alphaproteobacteria	Rhodoplanes sp Z2 YC6860	GCA_001579845.1
AMN43372.1_4907	RHPLAN_49480	Alphaproteobacteria	Rhodoplanes sp Z2 YC6860	GCA_001579845.1
AMN44490.1_6025	RHPLAN_60790	Alphaproteobacteria	Rhodoplanes sp Z2 YC6860	GCA_001579845.1
AMN41934.1_3469	RHPLAN_35020	Alphaproteobacteria	Rhodoplanes sp Z2 YC6860	GCA_001579845.1
AMN40143.1_1678	RHPLAN_16880	Alphaproteobacteria	Rhodoplanes sp Z2 YC6860	GCA_001579845.1
AMN41930.1_3465	RHPLAN_34980	Alphaproteobacteria	Rhodoplanes sp Z2 YC6860	GCA_001579845.1
AMN41688.1_3223	RHPLAN_32540	Alphaproteobacteria	Rhodoplanes sp Z2 YC6860	GCA_001579845.1
AMN44307.1_5842	RHPLAN_58960	Alphaproteobacteria	Rhodoplanes sp Z2 YC6860	GCA_001579845.1
AMN43220.1_4755	RHPLAN_47960	Alphaproteobacteria	Rhodoplanes sp Z2 YC6860	GCA_001579845.1
AMN43926.1_5461	RHPLAN_55100	Alphaproteobacteria	Rhodoplanes sp Z2 YC6860	GCA_001579845.1
AMN40896.1_2431	RHPLAN_24580	Alphaproteobacteria	Rhodoplanes sp Z2 YC6860	GCA_001579845.1
AMN41591.1_3126	RHPLAN_31560	Alphaproteobacteria	Rhodoplanes sp Z2 YC6860	GCA_001579845.1
AMN40096.1_1631	RHPLAN_16410	Alphaproteobacteria	Rhodoplanes sp Z2 YC6860	GCA_001579845.1
AMN40527.1_2062	RHPLAN_20870	Alphaproteobacteria	Rhodoplanes sp Z2 YC6860	GCA_001579845.1
AMN39130.1_665	RHPLAN_06680	Alphaproteobacteria	Rhodoplanes sp Z2 YC6860	GCA_001579845.1
AMN39351.1_886	RHPLAN_08890	Alphaproteobacteria	Rhodoplanes sp Z2 YC6860	GCA_001579845.1
AMN40827.1_2362	RHPLAN_23870	Alphaproteobacteria	Rhodoplanes sp Z2 YC6860	GCA_001579845.1
AMN41505.1_3040	RHPLAN_30690	Alphaproteobacteria	Rhodoplanes sp Z2 YC6860	GCA_001579845.1
AMN38614.1_149	RHPLAN_01490	Alphaproteobacteria	Rhodoplanes sp Z2 YC6860	GCA_001579845.1

AMN42077.1_3612	RHPLAN_36450	Alphaproteobacteria	Rhodoplanes sp Z2 YC6860	GCA_001579845.1
AMN44278.1_5813	RHPLAN_58670	Alphaproteobacteria	Rhodoplanes sp Z2 YC6860	GCA_001579845.1
AMN43333.1_4868	RHPLAN_49090	Alphaproteobacteria	Rhodoplanes sp Z2 YC6860	GCA_001579845.1
AMN43680.1_5215	RHPLAN_52610	Alphaproteobacteria	Rhodoplanes sp Z2 YC6860	GCA_001579845.1
AMN42076.1_3611	RHPLAN_36440	Alphaproteobacteria	Rhodoplanes sp Z2 YC6860	GCA_001579845.1
AMN40506.1_2041	RHPLAN_20660	Alphaproteobacteria	Rhodoplanes sp Z2 YC6860	GCA_001579845.1
AMN45246.1_6781	RHPLAN_68400	Alphaproteobacteria	Rhodoplanes sp Z2 YC6860	GCA_001579845.1
AMN41026.1_2561	RHPLAN_25880	Alphaproteobacteria	Rhodoplanes sp Z2 YC6860	GCA_001579845.1
AMN44481.1_6016	RHPLAN_60700	Alphaproteobacteria	Rhodoplanes sp Z2 YC6860	GCA_001579845.1
AMN44649.1_6184	RHPLAN_62380	Alphaproteobacteria	Rhodoplanes sp Z2 YC6860	GCA_001579845.1
AMN44187.1_5722	RHPLAN_57730	Alphaproteobacteria	Rhodoplanes sp Z2 YC6860	GCA_001579845.1
AMN44576.1_6111	RHPLAN_61650	Alphaproteobacteria	Rhodoplanes sp Z2 YC6860	GCA_001579845.1
AMN41487.1_3022	RHPLAN_30510	Alphaproteobacteria	Rhodoplanes sp Z2 YC6860	GCA_001579845.1
AMN39854.1_1389	RHPLAN_13990	Alphaproteobacteria	Rhodoplanes sp Z2 YC6860	GCA_001579845.1
AMN42294.1_3829	RHPLAN_38620	Alphaproteobacteria	Rhodoplanes sp Z2 YC6860	GCA_001579845.1
AMN44577.1_6112	RHPLAN_61660	Alphaproteobacteria	Rhodoplanes sp Z2 YC6860	GCA_001579845.1
AMN44470.1_6005	RHPLAN_60590	Alphaproteobacteria	Rhodoplanes sp Z2 YC6860	GCA_001579845.1
AMN42219.1_3754	RHPLAN_37870	Alphaproteobacteria	Rhodoplanes sp Z2 YC6860	GCA_001579845.1
AMN39816.1_1351	RHPLAN_13610	Alphaproteobacteria	Rhodoplanes sp Z2 YC6860	GCA_001579845.1
AMN39276.1_811	RHPLAN_08140	Alphaproteobacteria	Roseomonas gilardii	GCA_001941945.1
AMN42163.1_3698	RHPLAN_37310	Alphaproteobacteria	Roseomonas gilardii	GCA_001941945.1
AMN44378.1_5913	RHPLAN_59670	Alphaproteobacteria	Sinorhizobium meliloti 1021	GCA_000006965.1
AMN45183.1_6718	RHPLAN_67770	Betaproteobacteria	Achromobacter denitrificans	GCA_001514355.1
AMN41954.1_3489	RHPLAN_35220	Betaproteobacteria	Achromobacter insolitus	GCA_001971645.1
AMN40524.1_2059	RHPLAN_20840	Betaproteobacteria	Achromobacter insolitus	GCA_001971645.1
AMN45251.1_6786	RHPLAN_68450	Betaproteobacteria	Achromobacter xylosoxidans A8	GCA_000165835.1
AMN44857.1_6392	RHPLAN_64510	Betaproteobacteria	Achromobacter xylosoxidans A8	GCA_000165835.1
AMN43879.1_5414	RHPLAN_54630	Betaproteobacteria	Achromobacter xylosoxidans A8	GCA_000165835.1
AMN43409.1_4944	RHPLAN_49850	Betaproteobacteria	Achromobacter xylosoxidans A8	GCA_000165835.1
AMN40630.1_2165	RHPLAN_21900	Betaproteobacteria	Advenella kashmirensis WT001	GCA_000219915.3
AMN44639.1_6174	RHPLAN_62280	Betaproteobacteria	Advenella kashmirensis WT001	GCA_000219915.3
AMN40991.1_2526	RHPLAN_25530	Betaproteobacteria	Alicycliphilus denitrificans BC	GCA_000179015.2

AMN41886.1_3421	RHPLAN_34540	Betaproteobacteria	Alicycliphilus denitrificans BC	GCA_000179015.2
AMN41645.1_3180	RHPLAN_32100	Betaproteobacteria	Alicycliphilus denitrificans BC	GCA_000179015.2
AMN41522.1_3057	RHPLAN_30860	Betaproteobacteria	Alicycliphilus denitrificans BC	GCA_000179015.2
AMN43548.1_5083	RHPLAN_51250	Betaproteobacteria	Alicycliphilus denitrificans BC	GCA_000179015.2
AMN40093.1_1628	RHPLAN_16380	Betaproteobacteria	Bordetella bronchialis	GCA_001676705.1
AMN44321.1_5856	RHPLAN_59100	Betaproteobacteria	Bordetella bronchialis	GCA_001676705.1
AMN44384.1_5919	RHPLAN_59730	Betaproteobacteria	Bordetella bronchialis	GCA_001676705.1
AMN38627.1_162	RHPLAN_01620	Betaproteobacteria	Bordetella bronchialis	GCA_001676705.1
AMN41025.1_2560	RHPLAN_25870	Betaproteobacteria	Bordetella bronchialis	GCA_001676705.1
AMN42712.1_4247	RHPLAN_42820	Betaproteobacteria	Bordetella bronchiseptica RB50	GCA_000195675.1
AMN39602.1_1137	RHPLAN_11400	Betaproteobacteria	Bordetella flabilis	GCA_001676725.1
AMN41033.1_2568	RHPLAN_25950	Betaproteobacteria	Bordetella flabilis	GCA_001676725.1
AMN44911.1_6446	RHPLAN_65050	Betaproteobacteria	Bordetella flabilis	GCA_001676725.1
AMN45070.1_6605	RHPLAN_66640	Betaproteobacteria	Bordetella flabilis	GCA_001676725.1
AMN43555.1_5090	RHPLAN_51320	Betaproteobacteria	Bordetella flabilis	GCA_001676725.1
AMN43263.1_4798	RHPLAN_48390	Betaproteobacteria	Bordetella flabilis	GCA_001676725.1
AMN41861.1_3396	RHPLAN_34270	Betaproteobacteria	Bordetella flabilis	GCA_001676725.1
AMN41028.1_2563	RHPLAN_25900	Betaproteobacteria	Bordetella flabilis	GCA_001676725.1
AMN42047.1_3582	RHPLAN_36150	Betaproteobacteria	Bordetella genomosp 13	GCA_002119665.1
AMN44671.1_6206	RHPLAN_62600	Betaproteobacteria	Bordetella genomosp 13	GCA_002119665.1
AMN41520.1_3055	RHPLAN_30840	Betaproteobacteria	Bordetella genomosp 13	GCA_002119665.1
AMN43575.1_5110	RHPLAN_51520	Betaproteobacteria	Bordetella genomosp 13	GCA_002119665.1
AMN42001.1_3536	RHPLAN_35690	Betaproteobacteria	Bordetella genomosp 13	GCA_002119665.1
AMN41904.1_3439	RHPLAN_34720	Betaproteobacteria	Bordetella genomosp 13	GCA_002119665.1
AMN42443.1_3978	RHPLAN_40110	Betaproteobacteria	Bordetella genomosp 13	GCA_002119665.1
AMN40533.1_2068	RHPLAN_20930	Betaproteobacteria	Bordetella hinzii	GCA_001078275.1
AMN42645.1_4180	RHPLAN_42140	Betaproteobacteria	Bordetella hinzii	GCA_001078275.1
AMN39095.1_630	RHPLAN_06330	Betaproteobacteria	Bordetella hinzii	GCA_001078275.1
AMN45133.1_6668	RHPLAN_67270	Betaproteobacteria	Bordetella hinzii	GCA_001078275.1
AMN43571.1_5106	RHPLAN_51480	Betaproteobacteria	Bordetella hinzii	GCA_001078275.1
AMN44641.1_6176	RHPLAN_62300	Betaproteobacteria	Bordetella parapertussis 12822	GCA_000195695.1
AMN38525.1_60	RHPLAN_00600	Betaproteobacteria	Bordetella pertussis B1917	GCA_000193595.3

AMN42296.1_3831	RHPLAN_38640	Betaproteobacteria	Bordetella pertussis B1917	GCA_000193595.3
AMN41720.1_3255	RHPLAN_32860	Betaproteobacteria	Bordetella petrii	GCA_000067205.1
AMN43579.1_5114	RHPLAN_51560	Betaproteobacteria	Bordetella petrii	GCA_000067205.1
AMN40625.1_2160	RHPLAN_21850	Betaproteobacteria	Bordetella pseudohinzii	GCA_001698185.1
AMN45341.1_6876	RHPLAN_69350	Betaproteobacteria	Bordetella pseudohinzii	GCA_001698185.1
AMN40818.1_2353	RHPLAN_23780	Betaproteobacteria	Bordetella sp H567	GCA_001704295.1
AMN39384.1_919	RHPLAN_09220	Betaproteobacteria	Bordetella sp H567	GCA_001704295.1
AMN42752.1_4287	RHPLAN_43230	Betaproteobacteria	Bordetella sp H567	GCA_001704295.1
AMN38563.1_98	RHPLAN_00980	Betaproteobacteria	Bordetella sp H567	GCA_001704295.1
AMN42005.1_3540	RHPLAN_35730	Betaproteobacteria	Bordetella sp H567	GCA_001704295.1
AMN39912.1_1447	RHPLAN_14570	Betaproteobacteria	Bordetella sp H567	GCA_001704295.1
AMN43809.1_5344	RHPLAN_53930	Betaproteobacteria	Bordetella sp H567	GCA_001704295.1
AMN39953.1_1488	RHPLAN_14980	Betaproteobacteria	Bordetella trematum	GCA_900078695.1
AMN38654.1_189	RHPLAN_01890	Betaproteobacteria	Cupriavidus basilensis	GCA_000832305.1
AMN40586.1_2121	RHPLAN_21460	Betaproteobacteria	Cupriavidus basilensis	GCA_000832305.1
AMN44254.1_5789	RHPLAN_58410	Betaproteobacteria	Cupriavidus basilensis	GCA_000832305.1
AMN41157.1_2692	RHPLAN_27200	Betaproteobacteria	Cupriavidus basilensis	GCA_000832305.1
AMN41274.1_2809	RHPLAN_28370	Betaproteobacteria	Cupriavidus basilensis	GCA_000832305.1
AMN44946.1_6481	RHPLAN_65400	Betaproteobacteria	Cupriavidus basilensis	GCA_000832305.1
AMN39885.1_1420	RHPLAN_14300	Betaproteobacteria	Cupriavidus gilardii CR3	GCA_001281465.1
AMN38535.1_70	RHPLAN_00700	Betaproteobacteria	Cupriavidus gilardii CR3	GCA_001281465.1
AMN41132.1_2667	RHPLAN_26950	Betaproteobacteria	Cupriavidus gilardii CR3	GCA_001281465.1
AMN42803.1_4338	RHPLAN_43740	Betaproteobacteria	Cupriavidus metallidurans CH34	GCA_000196015.1
AMN40828.1_2363	RHPLAN_23880	Betaproteobacteria	Cupriavidus metallidurans CH34	GCA_000196015.1
AMN42079.1_3614	RHPLAN_36470	Betaproteobacteria	Cupriavidus necator N 1	GCA_000219215.1
AMN44661.1_6196	RHPLAN_62500	Betaproteobacteria	Cupriavidus necator N 1	GCA_000219215.1
AMN45295.1_6830	RHPLAN_68890	Betaproteobacteria	Cupriavidus sp USMAHM13	GCA_001854285.1
AMN43343.1_4878	RHPLAN_49190	Betaproteobacteria	Cupriavidus sp USMAHM13	GCA_001854285.1
AMN41496.1_3031	RHPLAN_30600	Betaproteobacteria	Delftia acidovorans SPH 1	GCA_000018665.1
AMN41383.1_2918	RHPLAN_29460	Betaproteobacteria	Diaphorobacter polyhydroxybutyrativorans	GCA_002214645.1
AMN41828.1_3363	RHPLAN_33940	Betaproteobacteria	Diaphorobacter polyhydroxybutyrativorans	GCA_002214645.1
AMN44286.1_5821	RHPLAN_58750	Betaproteobacteria	Diaphorobacter polyhydroxybutyrativorans	GCA_002214645.1

AMN45245.1_6780	RHPLAN_68390	Betaproteobacteria	Hydrogenophaga sp PBC	GCA_000263795.2
AMN41509.1_3044	RHPLAN_30730	Betaproteobacteria	Hydrogenophaga sp PBC	GCA_000263795.2
AMN39899.1_1434	RHPLAN_14440	Betaproteobacteria	Hydrogenophaga sp PBC	GCA_000263795.2
AMN43882.1_5417	RHPLAN_54660	Betaproteobacteria	Janthinobacterium sp Marseille	GCA_000013625.1
AMN44377.1_5912	RHPLAN_59660	Betaproteobacteria	Janthinobacterium sp Marseille	GCA_000013625.1
AMN40990.1_2525	RHPLAN_25520	Betaproteobacteria	Leptothrix cholodnii SP 6	GCA_000019785.1
AMN41811.1_3346	RHPLAN_33770	Betaproteobacteria	Limnohabitans sp 63ED37 2	GCA_001412535.1
AMN44191.1_5726	RHPLAN_57770	Betaproteobacteria	Polaromonas naphthalenivorans CJ2	GCA_000015505.1
AMN40381.1_1916	RHPLAN_19300	Betaproteobacteria	Polaromonas sp JS666	GCA_000013865.1
AMN44504.1_6039	RHPLAN_60930	Betaproteobacteria	Polaromonas sp JS666	GCA_000013865.1
AMN45121.1_6656	RHPLAN_67150	Betaproteobacteria	Polaromonas sp JS666	GCA_000013865.1
AMN38975.1_510	RHPLAN_05100	Betaproteobacteria	Polaromonas sp JS666	GCA_000013865.1
AMN41222.1_2757	RHPLAN_27850	Betaproteobacteria	Polaromonas sp JS666	GCA_000013865.1
AMN41255.1_2790	RHPLAN_28180	Betaproteobacteria	Ralstonia eutropha H16	GCA_000009285.2
AMN39603.1_1138	RHPLAN_11410	Betaproteobacteria	Ramlibacter tataouinensis TTB310	GCA_000215705.1
AMN38595.1_130	RHPLAN_01300	Betaproteobacteria	Ramlibacter tataouinensis TTB310	GCA_000215705.1
AMN41781.1_3316	RHPLAN_33470	Betaproteobacteria	Rhodoferax saidenbachensis	GCA_001955715.1
AMN44203.1_5738	RHPLAN_57890	Betaproteobacteria	Rhodoferax sp DCY110	GCA_001955695.1
AMN42036.1_3571	RHPLAN_36040	Betaproteobacteria	Rubrivivax gelatinosus IL144	GCA_000284255.1
AMN40411.1_1946	RHPLAN_19600	Betaproteobacteria	Variovorax sp PAMC 28711	GCA_001577265.1
AMN42229.1_3764	RHPLAN_37970	Betaproteobacteria	Verminephrobacter eiseniae EF01 2	GCA_000015565.1
AMN41135.1_2670	RHPLAN_26980	Betaproteobacteria	Verminephrobacter eiseniae EF01 2	GCA_000015565.1
AMN40341.1_1876	RHPLAN_18900	Deltaproteobacteria	Desulfococcus oleovorans Hxd3	GCA_000018405.1
AMN41462.1_2997	RHPLAN_30260	Deltaproteobacteria	Desulfococcus oleovorans Hxd3	GCA_000018405.1
AMN41795.1_3330	RHPLAN_33610	Gammaproteobacteria	Alcanivorax dieselolei B5	GCA_000300005.1

(TTT) family in the genome of the α-proteobacterium Rhodoplanes sp. Z2-YC6860"
ferent species. The first two rows refer to the analysis query, while the remaining relate to the analysis result

hit (e-value threshold = 10e-15)

Assembly name	Sequence ID	Identity (%)	Alignment length	Number of mismatches	Number of gap openings	E-value	Bit score
ASM156225v1	AMJ60317.1_1677	62.0	308	117	0	1.86E-143	405
ASM1136v1	BAC52326.1_7061	56.9	304	131	0	3.79E-127	364
ASM1136v1	BAC51735.1_6470	54.0	313	144	0	6.33E-112	326
ASM169338v1	ANW00367.1_1886	75.5	323	79	0	9.33E-177	490
ASM169338v1	ANW05692.1_3832	63.7	317	113	1	3.76E-150	422
ASM169338v1	ANW05692.1_3832	56.6	316	135	2	2.43E-127	365
ASM169338v1	ANW05692.1_3832	56.0	325	140	2	1.08E-131	375
ASM169338v1	ANW00602.1_2169	56.0	327	143	1	1.7E-141	401
ASM169338v1	ANW05692.1_3832	55.4	323	144	0	4.85E-138	392
ASM169338v1	ANW05692.1_3832	55.4	325	141	3	5.7E-128	366
ASM169338v1	ANW00602.1_2169	54.9	326	147	0	7.39E-140	396
ASM169338v1	ANW04363.1_6694	51.8	311	148	1	8.37E-118	340
ASM169338v1	ANW05692.1_3832	51.7	319	154	0	1.41E-120	348
ASM169338v1	ANV99810.1_1208	51.6	304	141	3	2.7E-104	306
ASM169338v1	ANW01425.1_3154	51.3	320	155	1	1.37E-119	345
ASM169338v1	ANV99613.1_966	49.8	297	147	2	1.6E-93	278
ASM169338v1	ANW05692.1_3832	46.8	327	168	5	8.02E-103	302
ASM169338v1	ANV99134.1_384	43.2	324	173	4	5.98E-78	239
ASM28437v1	BAL06143.1_842	64.3	325	116	0	1.62E-151	426
ASM28437v1	BAL07891.1_2590	47.0	319	165	2	3.69E-95	283
ASM28437v1	BAL07891.1_2590	44.2	319	173	3	1.24E-85	258
ASM34480v1	BAM90451.1_4430	71.7	325	88	2	8.69E-171	475
ASM34480v1	BAM91964.1_5943	56.2	317	139	0	7.96E-129	368
ASM34480v1	BAM92304.1_6283	52.9	329	154	1	4.26E-123	354
ASM34480v1	BAM93235.1_7214	47.7	321	168	0	1.34E-106	312

ASM19274v1	ADZ70905.1_2475	51.9	289	139	0	1.12E-104	306
ASM211976v1	ARP99846.1_2517	79.6	313	64	0	0	536
ASM211976v1	ARP98885.1_1419	75.2	294	73	0	5.42E-157	440
ASM211976v1	ARP99412.1_2017	72.7	319	84	2	1.03E-158	450
ASM211976v1	ARQ00167.1_2888	71.4	304	87	0	5.56E-164	458
ASM211976v1	ARP97840.1_228	69.9	306	92	0	2.3E-157	441
ASM211976v1	ARQ02181.1_5165	68.6	322	99	2	9.96E-161	449
ASM211976v1	ARP99484.1_2100	67.0	321	105	1	8.69E-149	419
ASM211976v1	ARQ00280.1_3024	66.2	302	102	0	6.22E-148	417
ASM211976v1	ARP99392.1_1993	65.3	323	108	2	1.1E-140	399
ASM211976v1	ARP98395.1_848	65.0	326	111	3	3.67E-142	402
ASM211976v1	ARP98001.1_417	64.5	324	114	1	1.56E-148	418
ASM211976v1	ARQ02450.1_5460	63.5	315	114	1	2.65E-150	424
ASM211976v1	ARQ00117.1_2827	63.2	299	107	3	1.62E-139	395
ASM211976v1	ARQ02236.1_5227	63.2	318	116	1	3.86E-152	428
ASM211976v1	ARQ01591.1_4486	62.8	304	113	0	6.69E-140	398
ASM211976v1	ARQ00364.1_3113	62.0	326	122	1	7.08E-147	414
ASM211976v1	ARQ01803.1_4738	60.3	310	123	0	1.48E-143	406
ASM211976v1	ARQ00280.1_3024	59.3	302	122	1	7.59E-126	362
ASM211976v1	ARP98858.1_1391	59.2	304	124	0	7.33E-139	394
ASM211976v1	ARQ00841.1_3650	58.9	319	131	0	2.69E-134	384
ASM211976v1	ARP98117.1_544	58.3	300	125	0	6.8E-123	353
ASM211976v1	ARQ02236.1_5227	57.8	320	131	3	1.5E-129	370
ASM211976v1	ARP98405.1_860	57.2	311	132	1	3.39E-129	369
ASM211976v1	ARQ02559.1_5583	57.1	319	136	1	6.82E-129	368
ASM211976v1	ARP97685.1_58	56.7	326	136	1	1.3E-145	411
ASM211976v1	ARQ02464.1_5476	56.7	312	135	0	2.53E-124	357
ASM211976v1	ARQ01810.1_4745	56.6	334	140	2	1.92E-126	363
ASM211976v1	ARQ00365.1_3114	56.2	297	129	1	4.77E-120	346
ASM211976v1	ARQ02066.1_5032	56.2	306	133	1	1.3E-121	350
ASM211976v1	ARP98405.1_860	56.2	331	139	2	2.58E-131	375
ASM211976v1	ARQ02126.1_5102	56.0	323	137	2	9.37E-131	373

ASM211976v1	ARQ00981.1_3805	54.8	292	130	2	1.53E-111	324
ASM211976v1	ARP98563.1_1048	54.7	298	134	1	3.86E-121	349
ASM211976v1	ARQ02236.1_5227	54.3	315	141	3	2.77E-123	355
ASM211976v1	ARQ01803.1_4738	54.2	319	145	1	1.16E-125	360
ASM211976v1	ARP99168.1_1733	54.1	320	145	1	2.39E-129	369
ASM211976v1	ARQ00365.1_3114	54.0	313	143	1	6.46E-121	348
ASM211976v1	ARQ01967.1_4916	53.9	317	145	1	2.75E-118	343
ASM211976v1	ARQ01182.1_4033	53.8	299	137	1	1.84E-111	324
ASM211976v1	ARP98405.1_860	53.6	330	148	2	2.32E-127	365
ASM211976v1	ARQ02041.1_5001	53.6	330	151	1	4.44E-127	364
ASM211976v1	ARQ00365.1_3114	53.6	289	129	3	6.53E-102	300
ASM211976v1	ARQ00365.1_3114	53.6	306	142	0	2.94E-113	328
ASM211976v1	ARQ00474.1_3237	52.4	315	149	1	8.09E-118	340
ASM211976v1	ARQ00111.1_2820	51.8	328	152	2	3.98E-119	345
ASM211976v1	ARQ00049.1_2747	51.7	325	152	1	3.49E-115	333
ASM211976v1	ARQ00981.1_3805	51.4	296	141	3	2.44E-105	308
ASM211976v1	ARQ02066.1_5032	51.3	302	144	2	4.25E-112	326
ASM211976v1	ARQ02236.1_5227	51.3	308	149	1	1.11E-116	338
ASM211976v1	ARQ00348.1_3096	50.8	297	146	0	3.07E-105	310
ASM211976v1	ARQ00075.1_2777	50.8	305	150	0	1.48E-115	335
ASM211976v1	ARQ02236.1_5227	50.3	332	158	3	7.08E-113	328
ASM211976v1	ARQ02236.1_5227	48.3	325	168	0	2.72E-116	337
ASM211976v1	ARQ02236.1_5227	48.3	321	165	1	3.01E-116	337
ASM211976v1	ARP98504.1_976	48.2	303	155	2	2.76E-95	286
ASM211976v1	ARQ00736.1_3532	47.7	323	165	2	7.02E-110	321
ASM211976v1	ARQ03119.1_4412	47.5	305	160	0	9.27E-99	292
ASM211976v1	ARQ02712.1_436	47.3	300	156	2	4.7E-93	277
ASM211976v1	ARP99168.1_1733	46.9	322	164	2	2.82E-103	303
ASM211976v1	ARQ02450.1_5460	46.9	318	166	3	1.17E-100	297
ASM211976v1	ARQ02126.1_5102	46.8	325	170	3	6.46E-107	313
ASM211976v1	ARP97693.1_66	46.7	317	164	2	1.32E-100	297
ASM211976v1	ARQ00075.1_2777	46.3	298	156	3	2.45E-93	278

ASM211976v1	ARQ00111.1_2820	46.2	318	169	2	1.17E-95	285
ASM211976v1	ARP97646.1_13	45.3	300	164	0	3.21E-88	266
ASM211976v1	ARQ02450.1_5460	44.9	305	167	1	4.63E-95	284
ASM211976v1	ARP99296.1_1886	43.9	319	176	2	4.08E-96	285
ASM211976v1	ARQ02126.1_5102	43.3	323	180	3	1.07E-92	276
ASM211976v1	ARQ01822.1_4757	43.2	303	169	2	7.34E-89	266
ASM211976v1	ARP99481.1_2097	42.9	317	175	3	1.03E-82	253
ASM211976v1	ARQ02450.1_5460	39.9	318	187	4	6.05E-77	237
ASM211976v1	ARP97824.1_211	37.3	308	188	2	2.43E-63	201
ASM157984v1	AMN41105.1_2640	80.1	322	63	1	0	513
ASM157984v1	AMN44660.1_6195	79.7	335	67	1	0	554
ASM157984v1	AMN44853.1_6388	78.1	329	72	0	0	509
ASM157984v1	AMN44679.1_6214	78.1	329	72	0	0	529
ASM157984v1	AMN45418.1_6953	74.2	325	84	0	0	512
ASM157984v1	AMN45417.1_6952	74.2	325	84	0	0	512
ASM157984v1	AMN45498.1_7033	73.6	326	86	0	0	505
ASM157984v1	AMN45497.1_7032	73.6	326	86	0	0	505
ASM157984v1	AMN39081.1_616	70.7	300	88	0	2.71E-150	423
ASM157984v1	AMN41292.1_2827	69.3	303	93	0	2.74E-157	441
ASM157984v1	AMN43264.1_4799	68.5	302	95	0	1.27E-157	441
ASM157984v1	AMN43565.1_5100	67.7	316	102	0	6.25E-152	427
ASM157984v1	AMN44369.1_5904	67.3	321	104	1	2.14E-155	436
ASM157984v1	AMN43897.1_5432	67.3	321	104	1	2.14E-155	436
ASM157984v1	AMN44552.1_6087	65.7	329	113	0	8.79E-154	432
ASM157984v1	AMN44387.1_5922	65.7	329	113	0	1.09E-155	437
ASM157984v1	AMN42088.1_3623	65.6	320	107	1	6.47E-158	442
ASM157984v1	AMN42620.1_4155	65.2	322	106	3	5.74E-153	429
ASM157984v1	AMN41292.1_2827	65.2	322	106	3	5.81E-153	429
ASM157984v1	AMN39298.1_833	65.1	301	103	2	6.12E-140	396
ASM157984v1	AMN42292.1_3827	64.3	305	108	1	9.08E-141	399
ASM157984v1	AMN41457.1_2992	64.3	305	108	1	9.22E-141	399
ASM157984v1	AMN39299.1_834	63.6	305	109	2	9.55E-141	399

ASM157984v1	AMN42082.1_3617	63.4	320	117	0	3.47E-140	397
ASM157984v1	AMN42083.1_3618	63.2	323	119	0	1.58E-145	410
ASM157984v1	AMN39857.1_1392	62.4	306	114	1	2.73E-132	377
ASM157984v1	AMN42802.1_4337	62.2	325	123	0	2.52E-146	413
ASM157984v1	AMN41822.1_3357	62.2	325	123	0	2.44E-146	413
ASM157984v1	AMN39858.1_1393	61.7	311	118	1	6.06E-135	384
ASM157984v1	AMN41455.1_2990	61.7	295	113	0	4.34E-138	392
ASM157984v1	AMN39623.1_1158	61.7	295	113	0	6.32E-138	391
ASM157984v1	AMN39337.1_872	61.1	301	116	1	2.56E-128	367
ASM157984v1	AMN45491.1_7026	60.5	324	126	1	8.32E-149	419
ASM157984v1	AMN44966.1_6501	60.5	324	126	1	8.4E-149	419
ASM157984v1	AMN40814.1_2349	60.5	311	121	1	7.99E-139	394
ASM157984v1	AMN40380.1_1915	60.5	311	121	1	8.57E-139	394
ASM157984v1	AMN43852.1_5387	60.3	315	124	1	4.15E-139	394
ASM157984v1	AMN45310.1_6845	60.1	308	123	0	5.86E-145	410
ASM157984v1	AMN42826.1_4361	60.1	308	123	0	1.01E-144	409
ASM157984v1	AMN42802.1_4337	59.2	326	133	0	9E-142	401
ASM157984v1	AMN41229.1_2764	58.5	299	123	1	3.55E-119	344
ASM157984v1	AMN42802.1_4337	58.5	323	134	0	9.83E-136	386
ASM157984v1	AMN45310.1_6845	58.4	305	127	0	2.57E-128	367
ASM157984v1	AMN41130.1_2665	58.0	324	131	3	1.18E-126	363
ASM157984v1	AMN39497.1_1032	58.0	324	131	3	2.44E-134	382
ASM157984v1	AMN43810.1_5345	57.9	302	127	0	2.11E-114	332
ASM157984v1	AMN41488.1_3023	57.9	304	128	0	3.89E-135	384
ASM157984v1	AMN40100.1_1635	57.9	304	128	0	4.85E-135	384
ASM157984v1	AMN44853.1_6388	57.8	320	132	1	6.36E-129	369
ASM157984v1	AMN40138.1_1673	57.8	320	132	2	2.71E-135	385
ASM157984v1	AMN44552.1_6087	57.7	319	132	2	4.02E-125	359
ASM157984v1	AMN38593.1_128	57.1	308	132	0	5.19E-132	377
ASM157984v1	AMN39299.1_834	57.1	308	128	1	2.06E-121	350
ASM157984v1	AMN43390.1_4925	57.0	298	127	1	1.92E-119	345
ASM157984v1	AMN44922.1_6457	56.7	300	130	0	9.21E-126	360

ASM157984v1	AMN40250.1_1785	56.6	304	127	1	6.5E-122	351
ASM157984v1	AMN41822.1_3357	56.5	322	138	2	2.38E-123	355
ASM157984v1	AMN41885.1_3420	56.5	322	139	1	5.49E-121	349
ASM157984v1	AMN41822.1_3357	56.4	314	137	0	1.05E-125	361
ASM157984v1	AMN39836.1_1371	56.3	302	131	1	2.92E-124	357
ASM157984v1	AMN41822.1_3357	56.3	318	137	2	1.69E-130	373
ASM157984v1	AMN42361.1_3896	56.3	304	132	1	6.69E-126	361
ASM157984v1	AMN40864.1_2399	56.2	297	130	0	5.11E-115	333
ASM157984v1	AMN41859.1_3394	56.1	301	131	1	5.19E-121	349
ASM157984v1	AMN39037.1_572	56.1	303	132	1	4.48E-125	359
ASM157984v1	AMN43514.1_5049	56.0	316	135	2	4.27E-117	338
ASM157984v1	AMN44921.1_6456	56.0	316	138	1	1.77E-128	367
ASM157984v1	AMN41394.1_2929	56.0	318	140	0	2.51E-136	387
ASM157984v1	AMN41041.1_2576	56.0	318	140	0	1.58E-131	375
ASM157984v1	AMN43389.1_4924	55.6	315	136	2	6.54E-120	346
ASM157984v1	AMN41040.1_2575	55.3	309	135	1	1.95E-124	357
ASM157984v1	AMN42802.1_4337	55.2	326	145	1	2.73E-131	375
ASM157984v1	AMN40364.1_1899	55.2	326	145	1	4.54E-129	369
ASM157984v1	AMN41040.1_2575	55.1	214	96	0	2.77E-88	261
ASM157984v1	AMN40540.1_2075	55.1	316	141	1	1.37E-126	362
ASM157984v1	AMN40100.1_1635	54.9	304	137	0	2.87E-126	362
ASM157984v1	AMN38592.1_127	54.8	332	145	1	2.81E-133	380
ASM157984v1	AMN38644.1_179	54.8	301	136	0	4.14E-114	331
ASM157984v1	AMN39129.1_664	54.7	322	146	0	5.66E-121	348
ASM157984v1	AMN41773.1_3308	54.7	322	143	2	1.12E-122	353
ASM157984v1	AMN39809.1_1344	54.6	326	144	1	7.57E-123	353
ASM157984v1	AMN44346.1_5881	54.5	321	142	2	4.28E-124	357
ASM157984v1	AMN40761.1_2296	54.5	321	142	2	4.08E-124	357
ASM157984v1	AMN40792.1_2327	54.2	312	141	2	5.6E-115	333
ASM157984v1	AMN40792.1_2327	54.1	320	144	2	1.23E-119	345
ASM157984v1	AMN41773.1_3308	54.0	322	143	2	1.02E-125	361
ASM157984v1	AMN39418.1_953	54.0	302	137	2	7.72E-113	328

ASM157984v1	AMN41662.1_3197	53.9	293	135	0	1.31E-116	336
ASM157984v1	AMN42802.1_4337	53.9	323	147	1	3.27E-126	362
ASM157984v1	AMN41586.1_3121	53.8	327	149	2	6.51E-116	336
ASM157984v1	AMN41856.1_3391	53.8	305	139	1	1.05E-115	335
ASM157984v1	AMN39809.1_1344	53.7	300	139	0	9.94E-108	315
ASM157984v1	AMN40138.1_1673	53.4	324	151	0	3.76E-124	357
ASM157984v1	AMN44952.1_6487	53.4	313	145	1	1.2E-117	340
ASM157984v1	AMN42861.1_4396	53.4	313	145	1	1.2E-117	340
ASM157984v1	AMN40381.1_1916	53.3	300	140	0	1.62E-119	345
ASM157984v1	AMN45416.1_6951	53.3	319	149	0	3.9E-120	347
ASM157984v1	AMN41662.1_3197	53.3	319	148	1	1.21E-120	348
ASM157984v1	AMN41267.1_2802	53.3	319	149	0	6.82E-123	354
ASM157984v1	AMN40935.1_2470	53.3	291	136	0	2.35E-113	330
ASM157984v1	AMN43567.1_5102	53.2	310	144	1	1.1E-117	342
ASM157984v1	AMN43839.1_5374	53.1	318	149	0	7.57E-116	335
ASM157984v1	AMN43823.1_5358	52.9	327	151	2	7.52E-115	333
ASM157984v1	AMN39510.1_1045	52.8	305	143	1	1.09E-117	340
ASM157984v1	AMN44609.1_6144	52.8	324	151	1	6.17E-124	357
ASM157984v1	AMN41728.1_3263	52.8	324	151	1	6E-118	341
ASM157984v1	AMN41822.1_3357	52.8	324	153	0	7.68E-121	348
ASM157984v1	AMN41488.1_3023	52.7	319	147	1	6.41E-121	349
ASM157984v1	AMN45418.1_6953	52.6	310	144	2	1.58E-111	325
ASM157984v1	AMN40381.1_1916	52.5	297	141	0	5.32E-116	336
ASM157984v1	AMN41394.1_2929	52.5	320	152	0	1.89E-121	350
ASM157984v1	AMN41396.1_2931	52.5	263	123	2	4.39E-97	285
ASM157984v1	AMN42545.1_4080	52.4	315	147	2	2.34E-113	329
ASM157984v1	AMN42646.1_4181	52.4	319	149	2	1.34E-111	326
ASM157984v1	AMN39299.1_834	52.3	302	144	0	3.02E-111	323
ASM157984v1	AMN39348.1_883	52.2	297	139	3	4.32E-106	310
ASM157984v1	AMN42083.1_3618	52.2	301	144	0	4.91E-112	326
ASM157984v1	AMN43364.1_4899	52.1	303	144	1	1.25E-109	319
ASM157984v1	AMN41039.1_2574	52.1	303	144	1	1.24E-109	319

ASM157984v1	AMN43183.1_4718	52.1	328	154	2	9.63E-120	345
ASM157984v1	AMN39036.1_571	52.1	328	154	2	1.86E-112	327
ASM157984v1	AMN41445.1_2980	52.0	298	143	0	1.02E-111	325
ASM157984v1	AMN45225.1_6760	51.8	301	144	1	2.47E-99	293
ASM157984v1	AMN39007.1_542	51.8	301	144	1	2.73E-106	311
ASM157984v1	AMN41394.1_2929	51.8	313	148	2	1.18E-113	330
ASM157984v1	AMN42450.1_3985	51.7	302	146	0	3.45E-112	328
ASM157984v1	AMN42147.1_3682	51.7	302	145	1	1.42E-110	322
ASM157984v1	AMN39966.1_1501	51.7	302	145	1	6.93E-102	300
ASM157984v1	AMN39592.1_1127	51.7	302	146	0	3.13E-112	328
ASM157984v1	AMN43568.1_5103	51.5	328	158	1	2.91E-114	333
ASM157984v1	AMN43536.1_5071	51.5	332	151	4	3.04E-106	311
ASM157984v1	AMN39534.1_1069	51.5	332	151	4	2.4E-103	304
ASM157984v1	AMN42424.1_3959	51.5	305	146	1	1.68E-113	330
ASM157984v1	AMN44921.1_6456	51.4	317	152	2	6.74E-120	346
ASM157984v1	AMN42646.1_4181	51.4	325	149	2	4.86E-114	332
ASM157984v1	AMN39243.1_778	51.4	325	149	2	4.34E-114	332
ASM157984v1	AMN43546.1_5081	51.2	324	155	3	4.61E-109	318
ASM157984v1	AMN41061.1_2596	51.2	324	155	3	4.69E-109	318
ASM157984v1	AMN42083.1_3618	51.2	299	146	0	8.4E-112	325
ASM157984v1	AMN44346.1_5881	51.1	307	148	1	8.87E-120	345
ASM157984v1	AMN43897.1_5432	51.1	313	153	0	5.52E-114	331
ASM157984v1	AMN38592.1_127	51.1	323	154	2	3.85E-116	337
ASM157984v1	AMN40142.1_1677	51.1	325	159	0	9.09E-120	345
ASM157984v1	AMN43364.1_4899	50.9	320	155	2	8.1E-108	315
ASM157984v1	AMN40935.1_2470	50.9	291	143	0	5.31E-107	313
ASM157984v1	AMN42332.1_3867	50.9	293	139	2	9.43E-99	291
ASM157984v1	AMN41856.1_3391	50.8	303	149	0	6.52E-110	320
ASM157984v1	AMN39560.1_1095	50.8	305	149	1	3.12E-104	306
ASM157984v1	AMN43823.1_5358	50.8	327	161	0	1.18E-114	333
ASM157984v1	AMN42618.1_4153	50.8	327	161	0	1.2E-107	315
ASM157984v1	AMN42333.1_3868	50.7	296	141	2	8.74E-100	294

ASM157984v1	AMN40100.1_1635	50.6	312	149	4	1.82E-102	302
ASM157984v1	AMN42783.1_4318	50.6	318	154	2	8.36E-104	306
ASM157984v1	AMN44737.1_6272	50.6	330	158	3	3.26E-116	337
ASM157984v1	AMN44909.1_6444	50.5	307	151	1	8.74E-111	322
ASM157984v1	AMN40891.1_2426	50.3	294	146	0	2.95E-102	301
ASM157984v1	AMN43364.1_4899	50.3	322	156	2	1.5E-102	301
ASM157984v1	AMN41662.1_3197	50.2	319	158	1	2.79E-116	337
ASM157984v1	AMN40891.1_2426	50.0	298	149	0	1.3E-102	302
ASM157984v1	AMN43248.1_4783	50.0	294	146	1	4.42E-111	323
ASM157984v1	AMN43246.1_4781	50.0	298	148	1	7.77E-113	328
ASM157984v1	AMN38699.1_234	49.8	325	162	1	2.02E-102	301
ASM157984v1	AMN41394.1_2929	49.8	315	157	1	6.48E-120	346
ASM157984v1	AMN41517.1_3052	49.8	297	148	1	1.01E-100	297
ASM157984v1	AMN42361.1_3896	49.7	304	152	1	1.53E-110	322
ASM157984v1	AMN38644.1_179	49.5	301	152	0	1.01E-109	320
ASM157984v1	AMN39037.1_572	49.5	297	148	1	3.86E-105	308
ASM157984v1	AMN40300.1_1835	49.4	326	164	1	4.04E-112	326
ASM157984v1	AMN43183.1_4718	49.4	326	161	2	4.03E-112	326
ASM157984v1	AMN41858.1_3393	49.4	322	162	1	2.18E-105	309
ASM157984v1	AMN38788.1_323	49.4	322	162	1	1.36E-106	312
ASM157984v1	AMN43183.1_4718	49.4	320	160	2	1.48E-109	320
ASM157984v1	AMN43841.1_5376	49.2	327	160	3	9.13E-112	325
ASM157984v1	AMN40387.1_1922	49.2	327	160	3	9.02E-112	325
ASM157984v1	AMN44488.1_6023	48.9	321	159	2	1.51E-114	332
ASM157984v1	AMN39283.1_818	48.9	321	159	2	1.51E-114	332
ASM157984v1	AMN38699.1_234	48.8	301	152	2	1.18E-95	284
ASM157984v1	AMN39348.1_883	48.8	295	150	1	4.84E-96	285
ASM157984v1	AMN44555.1_6090	48.6	296	152	0	2.2E-104	306
ASM157984v1	AMN39786.1_1321	48.6	296	152	0	2.14E-104	306
ASM157984v1	AMN41394.1_2929	48.6	321	163	1	1.4E-111	325
ASM157984v1	AMN44283.1_5818	48.2	330	167	1	9.65E-103	303
ASM157984v1	AMN44660.1_6195	48.1	289	150	0	8.87E-99	294

ASM157984v1	AMN41445.1_2980	48.0	300	154	2	2.12E-100	296
ASM157984v1	AMN38593.1_128	47.9	307	157	2	3.54E-108	317
ASM157984v1	AMN39348.1_883	47.8	299	155	1	3.75E-101	298
ASM157984v1	AMN43248.1_4783	47.4	293	154	0	6.51E-101	298
ASM157984v1	AMN43823.1_5358	47.2	326	170	1	2.78E-104	306
ASM157984v1	AMN43565.1_5100	47.1	323	167	1	7.6E-109	318
ASM157984v1	AMN38644.1_179	46.9	294	156	0	6.15E-91	272
ASM157984v1	AMN39786.1_1321	46.9	320	167	2	2.34E-92	276
ASM157984v1	AMN44623.1_6158	46.9	303	161	0	9.5E-95	282
ASM157984v1	AMN43536.1_5071	46.8	329	167	5	2.36E-95	283
ASM157984v1	AMN39348.1_883	46.7	330	173	2	9.41E-98	290
ASM157984v1	AMN44283.1_5818	46.6	326	166	2	1.3E-100	297
ASM157984v1	AMN40143.1_1678	46.4	304	161	1	1.32E-99	294
ASM157984v1	AMN43841.1_5376	46.3	324	163	5	1.39E-107	314
ASM157984v1	AMN41935.1_3470	46.2	314	168	1	1.57E-94	282
ASM157984v1	AMN39037.1_572	46.1	297	159	1	2.31E-99	293
ASM157984v1	AMN41935.1_3470	45.9	296	160	0	3.09E-94	280
ASM157984v1	AMN40144.1_1679	45.7	317	170	1	2.09E-100	296
ASM157984v1	AMN39302.1_837	45.7	300	161	1	6.96E-91	271
ASM157984v1	AMN39470.1_1005	45.5	299	161	1	6.7E-94	279
ASM157984v1	AMN45417.1_6952	45.3	311	168	1	1.57E-99	294
ASM157984v1	AMN43183.1_4718	45.3	320	173	2	8.88E-103	302
ASM157984v1	AMN42783.1_4318	45.3	316	173	0	7.16E-90	271
ASM157984v1	AMN41591.1_3126	45.0	320	172	2	2.42E-86	261
ASM157984v1	AMN40896.1_2431	45.0	320	172	2	4.99E-96	286
ASM157984v1	AMN44322.1_5857	44.9	305	164	4	2.6E-90	270
ASM157984v1	AMN44001.1_5536	44.5	330	175	3	1.77E-93	280
ASM157984v1	AMN39351.1_886	44.5	290	161	0	1.48E-92	276
ASM157984v1	AMN39130.1_665	44.5	290	161	0	4.59E-82	249
ASM157984v1	AMN44756.1_6291	44.5	290	160	1	1.54E-89	267
ASM157984v1	AMN39786.1_1321	44.2	301	164	1	1.83E-87	266
ASM157984v1	AMN39302.1_837	44.1	311	172	2	1.29E-96	286

ASM157984v1	AMN44278.1_5813	43.7	325	182	1	4.46E-91	273
ASM157984v1	AMN42077.1_3612	43.7	325	182	1	2.44E-96	286
ASM157984v1	AMN39302.1_837	43.5	322	176	2	5.87E-92	275
ASM157984v1	AMN38614.1_149	43.3	300	169	1	3.53E-85	257
ASM157984v1	AMN39592.1_1127	43.1	313	177	1	1.51E-91	275
ASM157984v1	AMN39007.1_542	43.0	305	173	1	1.73E-85	258
ASM157984v1	AMN43536.1_5071	42.9	333	181	2	2.47E-90	271
ASM157984v1	AMN41496.1_3031	42.6	312	178	1	1.73E-84	256
ASM157984v1	AMN44649.1_6184	42.6	317	180	1	4E-89	268
ASM157984v1	AMN44481.1_6016	42.6	317	180	1	4.29E-89	268
ASM157984v1	AMN42783.1_4318	42.4	302	174	0	3.36E-80	246
ASM157984v1	AMN39308.1_843	42.3	333	182	4	4.52E-86	260
ASM157984v1	AMN41520.1_3055	42.2	320	184	1	1.58E-91	274
ASM157984v1	AMN39966.1_1501	42.1	299	172	1	3.63E-84	255
ASM157984v1	AMN44187.1_5722	41.8	311	178	1	1.17E-81	248
ASM157984v1	AMN39858.1_1393	41.6	303	176	1	3.01E-84	255
ASM157984v1	AMN42350.1_3885	41.3	298	174	1	3.05E-81	247
ASM157984v1	AMN44481.1_6016	39.0	246	150	0	1.4E-63	199
ASM157984v1	AMN39434.1_969	38.7	313	187	5	1.35E-64	204
ASM194194v1	APT55936.1_234	52.7	300	140	1	1.73E-106	312
ASM194194v1	APT56580.1_1010	39.2	337	189	7	1.19E-69	218
ASM696v1	CAC46347.1_1774	38.3	290	179	0	4E-76	234
ASM151435v1	ALX85037.1_3677	45.2	323	177	0	7.98E-100	295
ASM197164v1	APX75522.1_2381	45.5	314	162	1	7.05E-90	269
ASM197164v1	APX75522.1_2381	42.9	303	171	2	6.12E-74	229
ASM16583v1	ADP15522.1_2160	45.0	318	171	3	1.09E-90	272
ASM16583v1	ADP15522.1_2160	43.4	316	174	3	2.09E-87	263
ASM16583v1	ADP17649.1_4287	43.3	321	182	0	1.4E-91	273
ASM16583v1	ADP15892.1_2530	40.6	310	183	1	2.07E-73	228
ASM21991v3	AFK61589.1_851	47.4	302	158	1	7.26E-94	280
ASM21991v3	AFK64071.1_3333	40.2	323	185	4	1.46E-76	236
ASM17901v2	ADU99363.1_1576	51.9	316	148	2	4.51E-110	320

ASM17901v2	ADV01228.1_3440	47.8	301	155	2	1.1E-98	292
ASM17901v2	ADU97812.1_25	42.1	302	172	3	5.13E-75	231
ASM17901v2	ADV00940.1_3152	40.9	303	176	3	1.38E-77	238
ASM17901v2	ADU98330.1_543	39.3	303	179	4	2.52E-76	235
ASM167670v1	ANN65508.1_726	47.2	318	165	1	3.13E-108	316
ASM167670v1	ANN66288.1_1639	44.4	297	162	3	2.21E-79	242
ASM167670v1	ANN67162.1_2665	42.7	300	170	2	5.12E-87	262
ASM167670v1	ANN66091.1_1407	41.9	298	173	0	1.49E-83	253
ASM167670v1	ANN68175.1_3836	39.6	298	173	4	3.96E-69	216
ASM19567v1	CAE34693.1_4322	39.3	313	183	3	2.76E-78	239
ASM167672v1	ANN78731.1_3527	48.8	322	157	5	2.61E-93	278
ASM167672v1	ANN78365.1_3109	47.8	324	166	2	1.91E-103	304
ASM167672v1	ANN75934.1_251	45.0	311	171	0	2.55E-94	281
ASM167672v1	ANN78973.1_3814	43.9	321	178	2	2.31E-90	271
ASM167672v1	ANN75934.1_251	43.7	323	180	2	2.66E-90	271
ASM167672v1	ANN78416.1_3170	41.5	316	180	2	4.55E-81	247
ASM167672v1	ANN78627.1_3412	40.9	303	175	4	3.76E-71	221
ASM167672v1	ANN76139.1_497	38.5	325	193	5	3.43E-69	217
ASM211966v1	ARP96556.1_4067	47.3	313	163	2	8.32E-101	297
ASM211966v1	ARP95149.1_2498	43.9	321	178	2	3.99E-94	280
ASM211966v1	ARP96556.1_4067	43.9	303	170	0	1.37E-87	263
ASM211966v1	ARP96333.1_3814	43.7	318	173	2	2.73E-91	273
ASM211966v1	ARP95536.1_2920	43.6	305	167	3	3.77E-86	260
ASM211966v1	ARP96556.1_4067	42.9	308	174	2	9.11E-83	251
ASM211966v1	ARP96556.1_4067	42.0	319	179	2	6.05E-76	234
ASM107827v1	AKQ57903.1_4541	50.5	295	146	0	9.24E-110	320
ASM107827v1	AKQ56159.1_2797	46.6	326	170	3	3.25E-88	265
ASM107827v1	AKQ54555.1_1193	46.0	313	169	0	3.42E-98	291
ASM107827v1	AKQ54555.1_1193	40.8	321	185	4	5.89E-83	252
ASM107827v1	AKQ54555.1_1193	40.2	331	195	3	1.76E-71	223
ASM19569v1	CAE37573.1_2271	45.2	299	163	1	1.09E-85	259
ASM19359v3	AIW92810.1_2200	56.2	299	131	0	5.78E-126	362

ASM19359v3	AIW92089.1_1479	41.9	310	179	1	2.6E-86	260
ASM6720v1	CAP41074.1_743	42.4	297	169	2	4.28E-81	246
ASM6720v1	CAP43764.1_3433	41.7	302	176	0	1.27E-84	256
ASM169818v1	ANY17866.1_3817	45.6	296	161	0	3.15E-96	285
ASM169818v1	ANY16017.1_1760	45.6	316	171	1	9.83E-99	291
ASM170429v1	AOB33652.1_2246	54.8	310	140	0	2.48E-117	339
ASM170429v1	AOB33906.1_3936	51.7	300	144	1	9.21E-102	300
ASM170429v1	AOB33882.1_3763	48.3	298	153	1	5.1E-99	293
ASM170429v1	AOB31346.1_2365	48.3	315	160	3	2.95E-99	293
ASM170429v1	AOB31531.1_2591	47.1	329	169	3	9.05E-101	297
ASM170429v1	AOB33469.1_1240	46.0	291	156	1	8.41E-84	253
ASM170429v1	AOB33748.1_2738	40.5	301	179	0	1.36E-72	225
H044680328	SAI68954.1_1543	47.8	312	161	2	2.55E-99	293
ASM83230v1	AJG23237.1_5822	48.7	304	156	0	3.27E-106	311
ASM83230v1	AJG23237.1_5822	46.8	299	159	0	2.13E-103	304
ASM83230v1	AJG23237.1_5822	44.6	303	165	3	8.55E-85	257
ASM83230v1	AJG24295.1_6880	44.3	300	163	3	1.6E-80	245
ASM83230v1	AJG24295.1_6880	44.2	283	152	3	3.03E-74	229
ASM83230v1	AJG24295.1_6880	42.7	300	168	3	1.03E-77	238
ASM128146v1	ALD93439.1_4179	45.8	299	161	1	2.17E-82	251
ASM128146v1	ALD92916.1_3656	42.6	296	165	2	1.1E-76	235
ASM128146v1	ALD92916.1_3656	37.4	297	185	1	4.44E-59	190
ASM19601v1	ABF07709.1_848	44.9	325	176	3	1.63E-88	266
ASM19601v1	ABF07709.1_848	40.3	315	184	4	1.19E-78	241
ASM21921v1	AEI78014.1_2644	40.3	310	185	0	1.14E-84	256
ASM21921v1	AEI82812.1_7442	38.5	299	181	3	6.09E-65	206
ASM185428v1	AOZ01685.1_3945	49.8	301	151	0	1.13E-108	318
ASM185428v1	AOZ03543.1_6178	45.2	292	159	1	1.75E-85	258
ASM1866v1	ABX32686.1_39	43.4	318	179	1	1.01E-89	269
ASM221464v1	ASI69578.1_2780	43.6	298	164	3	1.85E-86	261
ASM221464v1	ASI69870.1_3101	42.6	298	170	1	1.64E-85	259
ASM221464v1	ASI69870.1_3101	42.2	296	168	3	1.53E-80	246

Hint_PBC	AOS80664.1_3503	51.1	305	143	3	1.14E-103	305
Hint_PBC	AOS81499.1_4430	37.5	312	189	4	7.68E-63	200
Hint_PBC	AOS81910.1_4887	35.3	306	197	1	4.35E-63	201
ASM1362v1	ABR89622.1_470	62.8	304	113	0	4.16E-136	387
ASM1362v1	ABR89757.1_1291	40.1	302	180	1	6.95E-73	226
ASM1978v1	ACB32994.1_718	51.5	326	157	1	1.76E-115	335
ASM141253v1	ALK89897.1_2653	47.7	300	157	0	1.3E-98	291
ASM1550v1	ABM40046.1_4719	41.2	301	176	1	5.65E-82	249
ASM1386v1	ABE43844.1_1877	54.6	304	137	1	2.48E-121	350
ASM1386v1	ABE42578.1_611	51.9	324	149	4	4.23E-115	333
ASM1386v1	ABE44216.1_2249	44.1	315	176	0	3.16E-95	283
ASM1386v1	ABE46273.1_4306	43.8	317	177	1	4.3E-89	267
ASM1386v1	ABE46979.1_5012	39.5	304	181	3	1.02E-76	235
ASM928v2	CAJ95007.1_3852	37.1	318	190	5	4.15E-73	227
ASM21570v1	AEG92762.1_1703	51.3	298	145	0	3.24E-110	322
ASM21570v1	AEG93472.1_2413	47.0	330	169	1	7.38E-105	308
ASM195571v1	APW43304.1_2498	40.9	323	186	2	8.51E-86	259
ASM195569v1	APW39675.1_4495	51.4	323	151	1	6.03E-111	323
ASM28425v1	BAL96432.1_3093	62.5	301	113	0	8.86E-132	376
ASM157726v1	AMM25888.1_3386	40.9	320	186	2	6.52E-87	262
ASM1556v1	ABM59508.1_3715	49.5	291	145	2	4.21E-94	280
ASM1556v1	ABM59508.1_3715	44.1	322	179	1	2.8E-100	296
ASM1840v1	ABW68105.1_2295	36.0	311	199	0	2.32E-64	204
ASM1840v1	ABW68105.1_2295	30.3	304	193	8	1.42E-34	127
ASM30000v1	AFT68689.1_400	40.5	309	179	4	6.75E-69	215

Suplementary Table 2 for the manuscript "Massive overrepresentation of solute binding proteins (SBPs) from the tripartite tricarboxylate transporter (TTT) f
Suplementary Table 2: Number of TBLASTN hits belonging to different species, from the analysis of the 434 TTT SBP from *Rhodoplanes sp* . Z2-YC6860

species	subclass	number of Rhodoplanes best hits
Rhodoplanes sp Z2 YC6860	Alphaproteobacteria	234
Pseudorhodoplanes sinuspersici	Alphaproteobacteria	72
Bradyrhizobium icense	Alphaproteobacteria	15
Bordetella flabilis	Betaproteobacteria	8
Bordetella genomosp 13	Betaproteobacteria	7
Bordetella sp H567	Betaproteobacteria	7
Cupriavidus basilensis	Betaproteobacteria	6
Alicycliphilus denitrificans BC	Betaproteobacteria	5
Bordetella bronchialis	Betaproteobacteria	5
Bordetella hinzii	Betaproteobacteria	5
Polaromonas sp JS666	Betaproteobacteria	5
Bradyrhizobium oligotrophicum S58	Alphaproteobacteria	4
Achromobacter xylosoxidans A8	Betaproteobacteria	4
Bradyrhizobium japonicum USDA 6	Alphaproteobacteria	3
Cupriavidus gilardii CR3	Betaproteobacteria	3
Diaphorobacter polyhydroxybutyrativorans	Betaproteobacteria	3
Hydrogenophaga sp PBC	Betaproteobacteria	3
Bradyrhizobium diazoefficiens USDA 110	Alphaproteobacteria	2
Roseomonas gilardii	Alphaproteobacteria	2
Achromobacter insolitus	Betaproteobacteria	2
Advenella kashmirensis WT001	Betaproteobacteria	2
Bordetella pertussis B1917	Betaproteobacteria	2
Bordetella petrii	Betaproteobacteria	2
Bordetella pseudohinzii	Betaproteobacteria	2
Cupriavidus metallidurans CH34	Betaproteobacteria	2
Cupriavidus necator N 1	Betaproteobacteria	2
Cupriavidus sp USMAHM13	Betaproteobacteria	2
Janthinobacterium sp Marseille	Betaproteobacteria	2
Ramlibacter tataouinensis TTB310	Betaproteobacteria	2

Verminephrobacter eiseniae EF01 2	Betaproteobacteria	2
Desulfococcus oleovorans Hxd3	Deltaproteobacteria	2
Bosea sp PAMC 26642	Alphaproteobacteria	1
Polymorphum gilvum SL003B 26A1	Alphaproteobacteria	1
Sinorhizobium meliloti 1021	Alphaproteobacteria	1
Achromobacter denitrificans	Betaproteobacteria	1
Bordetella bronchiseptica RB50	Betaproteobacteria	1
Bordetella parapertussis 12822	Betaproteobacteria	1
Bordetella trematum	Betaproteobacteria	1
Delftia acidovorans SPH 1	Betaproteobacteria	1
Leptothrix cholodnii SP 6	Betaproteobacteria	1
Limnohabitans sp 63ED37 2	Betaproteobacteria	1
Polaromonas naphthalenivorans CJ2	Betaproteobacteria	1
Ralstonia eutropha H16	Betaproteobacteria	1
Rhodoferax saidenbachensis	Betaproteobacteria	1
Rhodoferax sp DCY110	Betaproteobacteria	1
Rubrivivax gelatinosus IL144	Betaproteobacteria	1
Variovorax sp PAMC 28711	Betaproteobacteria	1
Alcanivorax dieselolei B5	Gammaproteobacteria	1

'amily in the genome of the α-proteobacterium *Rhodoplanes sp* . Z2-YC6860"
